# What we (don’t) know about parrot welfare: Finding welfare indicators through a systematic literature review

**DOI:** 10.1017/awf.2024.61

**Published:** 2024-12-05

**Authors:** Andrea Piseddu, Yvonne RA van Zeeland, Jean-Loup Rault

**Affiliations:** 1 Centre for Animal Nutrition and Welfare, University of Veterinary Medicine Vienna, Veterinaerplatz 1, 1210 Vienna, Austria; 2 Division of Zoological Medicine, Department of Clinical Sciences, Faculty of Veterinary Medicine, Utrecht University, Yalelaan 108, 3584 CM Utrecht, The Netherlands

**Keywords:** animal welfare, feasibility, *Psittaciformes*, validity, welfare assessment, welfare indicator

## Abstract

Parrots are popular companion animals but show prevalent and at times severe welfare issues. Nonetheless, there are no scientific tools available to assess parrot welfare. The aim of this systematic review was to identify valid and feasible outcome measures that could be used as welfare indicators for companion parrots. From 1,848 peer-reviewed studies retrieved, 98 met our inclusion and exclusion criteria (e.g. experimental studies, captive parrots). For each outcome collected, validity was assessed based on the statistical significance reported by the authors, as other validity parameters were rarely provided for evaluation. Feasibility was assigned by considering the need for specific instruments, veterinary-level expertise or handling the parrot. A total of 1,512 outcomes were evaluated, of which 572 had a significant *P*-value and were considered feasible. These included changes in behaviour (e.g. activity level, social interactions, exploration), body measurements (e.g. body weight, plumage condition) and abnormal behaviours, amongst others. Many physical and physiological parameters were identified that either require experimental validation, or veterinary-level skills and expertise, limiting their potential use by parrot owners themselves. Moreover, a high risk of bias undermined the internal validity of these outcomes, while a strong taxonomic bias, a predominance of studies on parrots in laboratories, and an underrepresentation of companion parrots jeopardised their external validity. These results provide a promising starting point for validating a set of welfare indicators in parrots.

## Introduction

Parrots have always fascinated human beings, influencing art, literature, and religion across centuries and continents (Boehrer 2010). Today, these birds, belonging to the order *Psittaciformes* (Gill *et al.* 2022), are one of the most popular companion animals after dogs and cats (Kidd & Kidd 1998; Meyers 1998; Anderson 2003; Engebretson 2006). Such popularity can be attributed to their bright and colourful plumages, and to their learning abilities which are comparable to those of human toddlers (Pepperberg & Funk 1990; Pepperberg 2009; Spierings & ten Cate 2016; Eggleston *et al.* 2022). Parrots, supported by large and neuron-rich forebrains (Emery 2006; Olkowicz *et al.* 2016), can use and even manufacture tools (Auersperg *et al.* 2011, 2012, 2018; Lambert *et al.* 2015), think economically (Laumer *et al.* 2016; Krasheninnikova *et al.* 2018), succeed in problem-solving, reasoning and planning tasks (Rössler & Auersperg 2022), and even remember their own past actions (Torres Ortiz *et al.* 2022), an important prerequisite for self-awareness. Parrots also show different kinds of social competences: they can co-operate during problem-solving tasks (Tebbich *et al.* 1996; Péron *et al.* 2011; Schwing *et al.* 2016, 2021), learn from conspecifics (Auersperg *et al.* 2014; Klump *et al.* 2021) and exhibit prosocial behaviours (Krasheninnikova *et al.* 2019; Brucks & von Bayern 2020; Laumer *et al.* 2021). Similar to humans and a few other species, they can learn and imitate sounds (Pepperberg 2009; Vernes *et al.* 2021), and synchronise their motor output on incoming rhythmic acoustic or visual information (Patel *et al.* 2009; Schachner *et al.* 2009; Hasegawa *et al.* 2011).

These characteristics render parrots valuable and desirable companion animals (Kidd & Kidd 1998; Anderson 2014). However, their high social needs and cognitive abilities, along with specific dietary and husbandry requirements, also render companion parrots prone to developing serious health and welfare issues in captivity. Some common welfare issues are nutritional deficiencies (e.g. hypocalcaemia, hypovitaminosis A), and associated pathologies (e.g. metabolic bone disease, egg binding, secondary infections), other (non-infectious) diseases (e.g. atherosclerosis, obesity), and development of fear-related, aggressive, stereotypic and/or self-injurious behaviours such as feather-damaging behaviour (Koski 2002; Engebretson 2006; Kalmar *et al.* 2010; Speer *et al.* 2016; Seibert 2020).

Experts from 51 different countries predict an increase in the trading of parrots due to their popularity (Ribeiro *et al.* 2019). This will likely have detrimental consequences for the conservation of this highly threatened taxon (Olah *et al.* 2016; IUCN 2024), but it also implies an increase of companion parrots population. Despite this prediction and the well-known welfare challenges of keeping captive parrots, there are currently no standardised guidelines for evaluating companion parrot welfare. Identifying welfare indicators would enable significant advancements to be made in companion parrot welfare, as they could be used for instantaneous assessments and in a repeated manner to monitor and evaluate changes in the parrots’ welfare. Additionally, it could increase a caregiver’s understanding of parrots’ needs, ultimately improving parrots’ overall quality of life. Suitable welfare indicators can be identified by following five key steps.

The first step is to find and collect information from peer-reviewed scientific studies. Integrating scientific information represents the most appropriate strategy, as it allows welfare assessors to employ standardised and objective methods, lowering the risk of making assessments biased by personal experience, mood, and emotional subjective states (Tuyttens *et al.* 2014; Mota-Rojas *et al.* 2021).

However, systematic reviews and simulations have shown that, due to weak experimental designs and settings, single research studies carry a high risk of bias, which is defined as “*a systematic error or deviation from the truth, in results or inferences*” (Boutron et al. 2019). For this reason, the second step to identify welfare indicators is to verify the internal validity of the scientific findings collected. Internal validity is defined as “*the extent to which the design and conduct of a study are likely to have prevented bias*” , and it is typically divided in four sub-categories: construct validity (i.e. the extent to which a test measures what it is intended to measure) (Cronbach & Meehl 1955), face validity (i.e. appropriateness of the test and its parameters) (Gravetter & Forzano 2012), content validity (i.e. the extent to which the test covers the entire construct) (Lawshe 1975), and criterion validity (i.e. the extent to which the outcomes of the test aligns with those previously obtained with validated instruments or “*gold standards*”) (Bellamy 2015). “*Internal validity*” and “*risk of bias*” are closely associated (Viswanathan *et al.* 2008); in fact, when a test presents high risk of bias, its results cannot be considered internally valid. Similarly, reliability, which is defined as production of consistent results within the same subject (“*test-retest reliability*”) (Gravetter & Forzano 2012), or between (“*inter-observer reliability*”) and within (“*intra-observer reliability*”) observers (Martin & Bateson 2007), is an important prerequisite for internal validity. A reliable measure is not necessarily valid; however, when the measure is not reliable, it cannot be valid (Gravetter & Forzano 2012). As such, it is important to screen scientific studies according to the aforementioned parameters to assess their internal validity.

The third step is to verify the studies’ external validity, i.e. the extent to which the findings of a study can be generalised and applied to other species, environmental conditions, or experimental settings (Lehner 1998; Bailoo *et al.* 2014). This is especially important in the case of parrots as the *Psittaciformes* order comprises a vast diversity of species. Unlike ‘dog’, ‘cat’ or ‘rabbit’, ‘parrot’ is a general term grouping more than 350 species that are adapted to different ecological niches and have distinct environmental, dietary, and behavioural needs (Bright-Smith 1999). This raises the question whether conclusions drawn from studies on a single species can be applied to other species (Hill & Broom 2009). For our purposes, it is necessary to determine whether and to what extent the species of interest, i.e. those commonly kept as companion animals, have been studied. Similarly, the setting in which the study results have been obtained should be considered as living conditions in a zoo, shelter or laboratory differ markedly from those in a private household, thereby implying that results obtained under these circumstances are not necessarily applicable to parrots kept as companions.

The fourth step is to identify feasible measurements. As suggested by Yon and colleagues (2019), animal welfare assessments should ideally be “*rapid, non-invasive and should not require any specialist equipment, facilities or specific training*”. This considerably reduces the risk of errors due to, for instance, assessors’ tiredness, instruments’ accuracy and sensitivity, or the animal’s reaction in response to handling.

According to Fraser (2008), animal welfare should be assessed by employing measurements that reflect three distinct inextricable conceptual frameworks: the animal’s affective state, its biological functioning, and natural living. As such, the fifth and final step requires capturing the various welfare dimensions through different indicators. Although behavioural indicators are considered the best reflection of animals’ ability to cope with their environment (Hill & Broom 2009), a more accurate welfare assessment can be obtained by combining behaviour with other measurements, including physical condition, physiological parameters, presence of disease and pathologies, husbandry, nutrition, and management considerations.

Given the current lack of science-based welfare indicators to evaluate the welfare of captive parrots, we conducted a systematic literature review in which we reframed the five key steps according to the following research questions:

- Which, if any, scientific results related to the welfare of captive parrots can be considered valid and feasible welfare indicators?
- How many and which types of welfare indicators have been identified?
- From how many and which parrot taxa have these indicators been collected? and
- How much and what type of information is available specifically regarding companion parrots?

Although our main target were companion parrots, we also collected information gathered from studies focused on other types of captive parrots, with the objective of identifying welfare indicators still applicable to our category of interest.

## Materials and methods

All phases of this study were conducted following the PRISMA 2020 statement for reporting systematic reviews (Page *et al.* 2021).

### Systematic search

A systematic search was conducted to find all scientific, peer-reviewed studies relevant to the research questions. The population, intervention, control, outcomes (‘PICO’) strategy (Nishikawa-Pacher 2022) was followed as much as possible and allowed to identify key terms related to the population of interest, to the type of intervention, and to the outcomes collected (note that the ‘control’ was not included as a search term as we did not restrict our search to case-control studies focusing on comparison of two interventions or comparison of the intervention with a control). We used terms such as ‘parrot’, ‘parakeet’, ‘psittacids’ and the specific type of parrot (e.g. macaw, grey parrot) for the population; terms related to nutrition, husbandry and management (e.g. ‘foraging enrichment’, ‘diet’, ‘hand-rearing’) for the intervention; and terms such as ‘abnormal behaviour’, ‘disease’, ‘life span’, ‘emotional state’, or specific behaviour problems, diseases or pathologies (e.g. ‘feather picking’, ‘atherosclerosis’, ‘obesity’) for the outcome (for the complete list of the 86 search key terms, see Table S1 in the Supplementary material). These key terms were then combined to create a search query using the Boolean operators AND, OR and NOT, and the queries were uploaded on the advanced search tool of the databases, PubMed, CAB Direct and Web of Science (on May 16^th^, 2022).

### Paper selection: Title and abstract screen, full-text screen

Following the literature search, the scientific studies found were uploaded into a reference manager (Endnote X7; The EndNoteTeam 2013). After removal of duplicates and triplicates, all remaining texts were screened twice using exclusion and inclusion criteria (Table S2; Supplementary material). Studies could involve parrots from any species, gender or age, and a variety of different interventions and outcomes (as specified in Table S2) and were included for further evaluation as long as the study was conducted in a captive population and was not focused on reproductive parameters (egg hatchability, number of eggs) or chick development as we considered these irrelevant for companion parrots. Further restrictions were related to language (English only), methodology (no studies involving < 5 subjects or without statistical analysis), publication type (full papers describing original research only) and retrievability of the paper. The first screening, based on the title and abstract, was conducted by one reviewer (AP), and aimed to exclude all studies that were considered irrelevant to address the research questions. All studies that passed this initial screening subsequently underwent a second screening, in which the full-text was read by two independent reviewers (AP, J-LR, see Supplementary material). Additional studies found through external sources (e.g. cited references) were also considered for their eligibility to ensure to encompass the latest literature available (updated until January 27^th^, 2023).

### Data collection

#### Collection of the outcome measures and corresponding risk factors

All behavioural, physiological, physical and health parameters that could potentially be linked to parrot welfare were collected from studies that passed the exclusion and inclusion criteria. Only parameters that were included as “*outcome measures*” (Percie du Sert *et* *al.* 2020), i.e. as part of the experimental design, were considered for further evaluation. For instance, outcome measures such as feeding behaviour or body weight were collected and considered only when measured specifically in relation to examined environmental conditions (e.g. social isolation, unbalanced diet, cage size or use of enrichment items) but not when correlated with natural biological phenomena (e.g. breeding season changes). To consider an outcome measure as a potential welfare indicator, a significant statistical link has to exist between this measure and the examined environmental conditions. A significant *P*-value in fact reflects that this outcome measure (e.g. behaviours, body measurement) is sensitive to a specific environmental condition that can therefore be considered a risk factor for parrot welfare (e.g. being housed alone vs in group, enrichment provided vs not provided). For this reason, we collected the *P*-value associated with each outcome measure and its corresponding risk factor. If not explicitly written by the authors, the potential risk factors were interpreted from the experimental conditions. For instance, in studies that compared behaviours of enriched and non-enriched parrots, the main risk factor for welfare was the lack of enrichment, in studies comparing the behaviours of hand-reared vs parent-reared subjects, the risk factor was being hand-reared, whereas for studies where parrots displayed a preference for a specific object or food item, the risk factor was a lack of offered choices. All risk factors associated with outcome measures with a *P*-value < 0.05 (both feasible and non-feasible) that were easy to define and identify (e.g. social isolation, diet, cage size etc) were also collected.

### Internal validity and feasibility of outcome measures

Aside from evaluating presence of a significant *P*-value, hence significant correlation between environmental parameter and outcome measure, outcome measures were also assessed for internal validity or risk of bias, which was a second criterion for assessing overall validity of welfare parameters. As multiple outcome measures could be identified from a single study, and because our focus was on identifying all outcome measures that could be used as parrot welfare indicators, we assessed the internal validity of each outcome measure separately rather than for the study as a whole, as is commonplace for most systematic reviews. For this purpose, we employed selected domains of the SYRCLE’s RoB tool (Hooijmans *et al.* 2014): from the main text, we extrapolated whether data were measured and collected by a blinded assessor, whether studied population was randomised (where applicable) and, in case of experimental set-ups with multiple conditions, whether the design was balanced between subjects and/or groups. In addition, we checked for tests of intra- and inter-observer reliability (see Table S3 in the Supplementary material for definitions of each of the evaluated validity parameters).

Given that we aimed to find welfare indicators that could be used to assess parrot welfare in practice, we also evaluated the outcome measures for their feasibility. They were classified as feasible if the behavioural assessment or measurements could be readily performed, requiring only the use of commonly available equipment (e.g. weight scale) or the use of minimally invasive routine handling techniques, or as non-feasible if the use of specific instrumentation, calculation or veterinarian-level skills or expertise were required.

### Grouping of outcome measures in welfare categories and welfare dimensions

To facilitate interpretability of the results and determine how many outcome measures of a certain type could be identified, these were first grouped in categories according to commonalities in their underlying biological construct (e.g. stereotypic behaviour, body condition, foraging behaviour). These categories were then classified into one of eight distinct welfare dimensions, which were created by grouping the welfare categories according to their shared characteristics. (i.e. physical or physiological measures, abnormal and fear-related behaviours, maintenance behaviours, locomotory behaviours, exploratory and foraging behaviours, social behaviours, and diseases and pathologic conditions, see Table S4; Supplementary material).

### Extrapolability of outcome measures across species and settings

To determine the extent to which outcome measures would be applicable or extrapolable to other species or settings, we collected taxonomic data (parrot species and genus), where available. Data acquired from multiple genera were classified under the category ‘multiple’. Following the definition of ‘pet’ or ‘companion’ animal proposed by Farnworth (2018), the studied subjects were identified as companion parrots when they “*lived with humans or within human social structures where they were provided with some, or all, of their needs*” and “*played a primarily social role within a household or community*”. Alternatively, we identified the parrots based on their living conditions as parrots kept in laboratories, zoos, shelters or breeding centres (see Table S5 in the Supplementary material for the complete list and definitions of subjects’ living conditions).

### Data analysis

Outcomes with a significant *P*-value (alpha threshold fixed at 0.05) were identified and subsequently screened for their feasibility. The outcome measures with a significant *P*-value and considered feasible were then grouped according to the welfare categories and dimensions they belonged, and according to subjects’ characteristics (genus and living condition). Data were analysed using the R statistical software (R CoreTeam 2022) and the R package ‘dplyr’ (Wickham 2023). Figures were created using the R package ‘ggplot2’ (Wickham 2016).

## Results

### Result of the systematic search and paper selection

**Figure 1.**
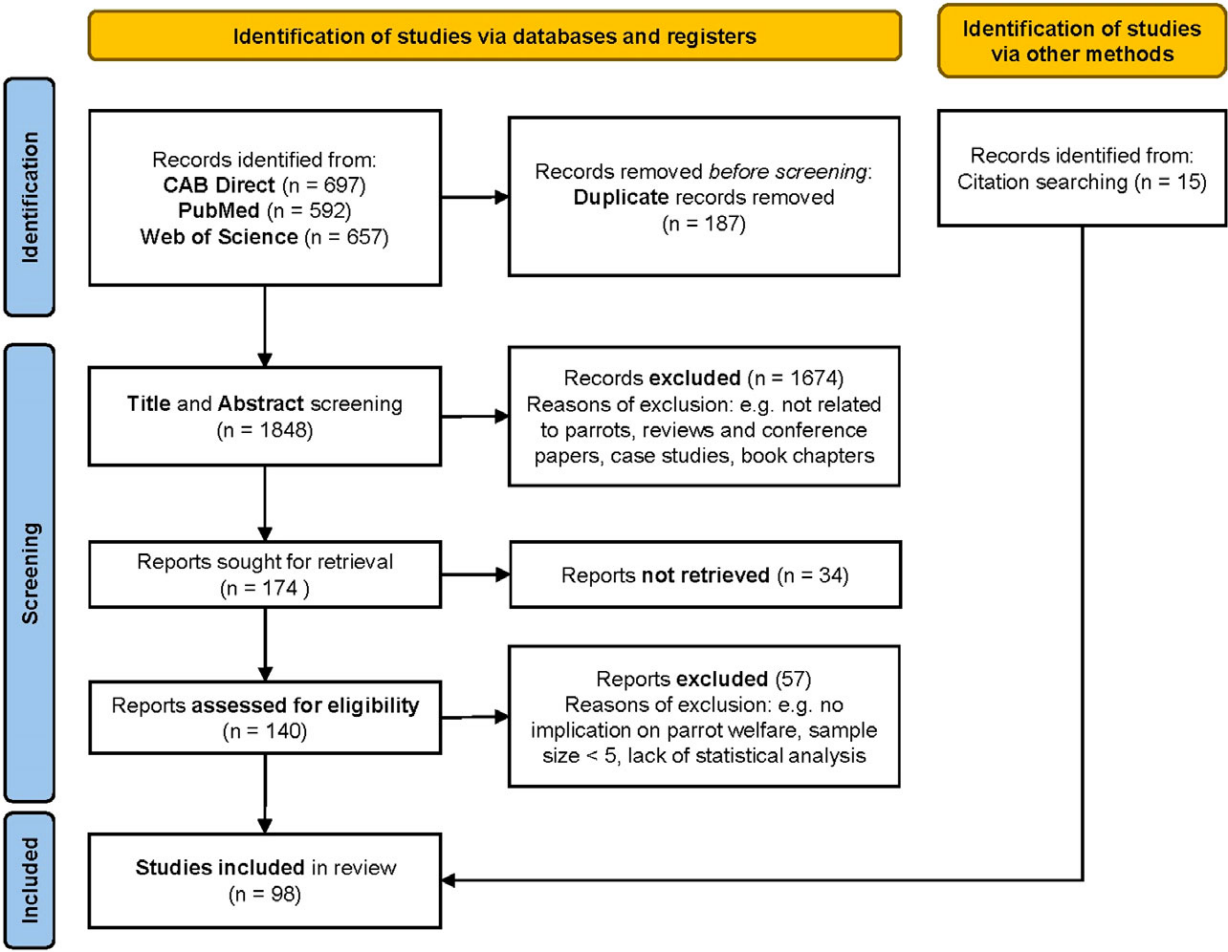
PRISMA flowchart identifying the number of studies reporting on parrot welfare parameters retrieved during the literature search from each database (PubMed, Web of Science, CAB Direct) and via other methods, the number of studies subjected to a first screening based on title and abstract reading and a second based on eligibility criteria, studies excluded during both screening phases, and the number of studies included in the final review.

The systematic search led to the collection of 1,946 scientific studies: 697 from CAB direct, 657 from Web of Science and 592 from PubMed. A total of 189 studies were found to be duplicates and triplicates and, after removing these, the total amount of hits dropped to 1,848. The first screening, based on title and abstract reading, led to the selection of 140 studies. The second screening, based on full-text reading, led to the retention of 83 studies. An additional 15 studies were found from external sources. This screening step led to a final amount of 98 studies from which data were collected (Figure 1).

### General results

The year of publication of eligible studies ranged from 1993 to 2023, with 76 studies (77.6%) published between 2010 and 2023. Of the studies that were eligible for full evaluation, 95 (96.9%) reported significant results and, of these, 72 (73.5%) reported outcome measures that were considered feasible to be carried out by owners (see Table S8; Supplementary material). Only 13 of these 72 studies with significant and feasible outcomes (13.3%) specifically related to companion parrots (see Table S8). The number of outcomes collected in a study ranged from 1 to 100 (median: 11). Of the total of 1,512 outcomes collected, 720 (47.6%) had a significant *P*-value, and of those 572 (37.8%) were also considered feasible. Of these 572 outcomes, 68 (4.49%) were obtained from companion parrots.

### Risk of bias

Intra- and inter-observer reliability and assessor blindness were reported for less than 5% of the outcome measures and were not specified for more than 77% of the outcomes (Table 1). In addition, a high number of outcome measures were from studies that, due to their experimental set-up, did not allow to control for the presence of biases or prevent it. For instance, outcomes collected from questionnaires and retrospective studies (17.5%) could not be tested for intra- and inter-observer reliability, and random assignment of subjects to groups, assessor blindness and balancing of experimental conditions could not be applied to this type of studies. Another example came from studies where subjects were assigned to control and enriched groups: in this case, it may not have been possible to blind the assessors and therefore to control for this specific bias. Due to these circumstances, we could not establish with certainty the internal validity of the findings collected from the studies.

**Table 1.** Assessment of the risk of bias for the outcome measures (n = 1,512) related to parrot welfare identified in the systematic literature search by using five validity parameters. The percentages refer to outcome measures for which the validity parameters, as indicated in the table, were reported by the authors (‘Yes’), were not considered by the author (‘No’), were not executable (‘Not possible’), or data regarding the validity parameter were not reported in the main text (‘Not specified’)

	Validity parameters				
Reporting	Inter-observer reliability	Intra-observer reliability	Random assignment of subjects to groups	Assessor blinded	Balancing of experimental conditions
Yes	2.9%	3.4%	17.9%	2.2%	15.7%
No	0%	0%	0%	1.1%	0.1%
Not possible	19.6%	19.5%	78.3%	63.6%	67.3%
Not specified	77.5%	77.1%	3.8%	33.1%	16.9%

### Representation of welfare dimensions and categories

#### Outcomes classified according to welfare dimensions

**Figure 2.**
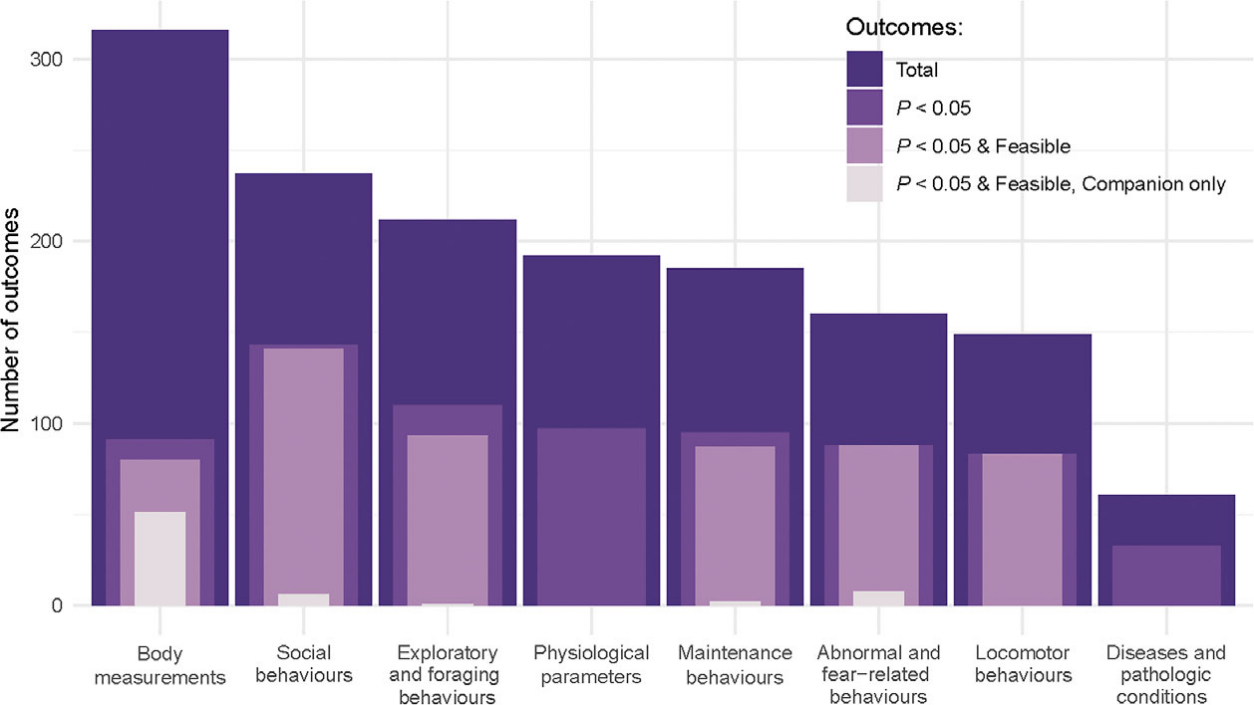
Number of outcomes related to parrot welfare as identified in the systematic literature search, grouped by welfare dimensions according to the biological construct that they represent. For each welfare dimension, the overlapped bar plot indicates, from darkest to lightest colour, the total number of outcome parameters collected, the number of significant outcomes (i.e. *P*-value < 0.05), the number of significant outcomes that are considered feasible for owners to assess (i.e. not requiring specific skills, expertise or equipment), and the number of significant and feasible outcomes collected from companion parrots (smallest bar).

Out of the eight welfare dimensions, the welfare dimension with the highest number of feasible and significant outcomes was ‘social behaviours’ (n = 141), whereas for most other dimensions the number of significant and feasible outcomes ranged between 80 and 93 (Figure 2, Table S9; Supplementary material). The welfare dimension ‘physiological parameters’ included a high number of significant outcomes (n = 97); however, all of these were considered not feasible as they required invasive sampling techniques (e.g. venipuncture) and laboratory equipment. A similar trend was noted for the welfare dimension ‘diseases and pathologic conditions’, albeit the number of outcomes reported was lower to start with (Figure 2).

### Outcome measures classified according to categories

The significant and feasible outcome measures were grouped in 26 different welfare categories (Table S10; Supplementary material). ‘Stereotypies’ covered the highest number of significant and feasible outcomes measures (n = 76) and captured oral stereotypies (e.g. wire or sham chewing), head stereotypies (e.g. spot pecking), and locomotor stereotypies (e.g. pacing, route tracing). ‘Indirect measures of feather-damaging behaviour’ included multiple scoring methods related to feather condition or feather improvement and was the category with the second highest number of significant and feasible outcome measures (n = 69), followed by ‘self-care’ (n = 42) with behaviours such as preening and stretching (Table S10). ‘Human-animal interaction’ was the category with the highest variety and included measures such as response to unfamiliar and familiar handlers, human-direct aggressiveness, and food acceptance (Table S10). Outcome measures, such as body weight and body mass, were grouped in the category ‘body condition’; walking, climbing, and flying in ‘locomotion’; food intake and feeding bout in ‘feeding’; crown, nape, cheek feather ruffling, crest erection and beak grinding in ‘facial and body displays’. Of the 68 significant and feasible outcomes measures collected from companion parrots, 51 referred to feather-damaging behaviour, five to human-animal interactions, five to stereotypies, three to fear-related behaviour, one to foraging behaviour, and one to sexual behaviour (Table S12; Supplementary material).

### Association between risk factors and outcomes measures

Significant and not feasible outcome measures were grouped in eight welfare categories and were related to various risk factors (Table S11; Supplementary material). For instance, human (neonatal) handling was linked to changes in the immune system (Collette *et al.* 2000), increased respiration rate (Aengus & Millam 1999) and increased serum corticosterone concentrations (Collette *et al.* 2000); social isolation negatively affected telomere length which has been associated with shorter lifespan (Aydinonat *et al.* 2014); and indoor housing and lack of UV-B lighting increased the risk for vitamin D deficiency (West *et al.* 2019; Nightengale *et al.* 2022). An unbalanced diet was correlated with changes in several parameters, including feather colour (crown feathers luminance) (Berg *et al.* 2019); immune system responses (increased haemoglobin, lymphocyte, monocyte and leucocyte counts, increased heterophil/lymphocyte ratio) (Berg *et al.* 2019; Di Santo *et al.* 2019); increased plasma, aortic, arterial and hepatic cholesterol levels (Beaufrere *et al.* 2013); changes in echocardiographic parameters associated with cardiovascular dysfunctions (Dos Santos *et al.* 2022); and increased incidence of atherosclerosis (Di Santo *et al.* 2019) (Table S11; Supplementary material).

Risk factors were also identified for significant and feasible outcome measures. For instance, the lack of environmental enrichment was associated with 18 welfare categories, including emergence of stereotypies and feather-damaging behaviours, decrease of physical activity and preening (Table S10; Supplementary material). Social isolation represented a risk factor for developing stereotypies (Williams *et al.* 2017), and was associated with reduced preening (Williams *et al.* 2017), flying (Nicol & Pope 1993) and locomotor activities (Meehan *et al.* 2003), and increased vocalisations (Nicol & Pope 1993) and avoidance behaviour towards humans (Meehan *et al.* 2003). Being hand-reared was correlated with the development of feather-damaging behaviour (Schmid *et al.* 2006; Costa *et al.* 2016), stereotypies (Williams *et al.* 2017) and preferential interactions with humans (Schmid *et al.* 2006) (Table S10). Living in a small cage was associated with the emergence of phobic behaviours (Schmid *et al.* 2006); abnormal behaviours such as incessant screaming, oral and locomotor stereotypies, increase of courtship behaviours and singing towards conspecifics (Polverino *et al.* 2015); and increase of locomotor activities and preening (Polverino *et al.* 2015; Phillips *et al.* 2018) (Table S10). Regarding the significant and feasible outcome measures collected from companion parrots, risk factors related to human-animal interactions (e.g. little time spent interacting each day, being hand-reared or wild caught, being acquired before the end of weaning or from a pet shop) were the most common investigated (Table S12; Supplementary material).

### Representation of the various genera and living conditions

#### Living conditions represented in the studies

The majority of studies (n = 48) were conducted on parrots kept in laboratories, followed by 22 on companion parrots, ten on parrots kept in zoos, six on parrots kept in breeding facilities, three on parrots kept in rehabilitation centres and two on parrots kept in shelters (Table S6; Supplementary material). Two studies focused on comparing parrots kept in different settings (i.e. wild versus companion parrots vs parrots kept in zoos or breeding facilities, and companion vs parrots kept in laboratories; Table S6). For seven studies, it was not possible to define the living condition of the parrots (Table S6).

### Outcome measures in relation to genera

**Figure 3.**
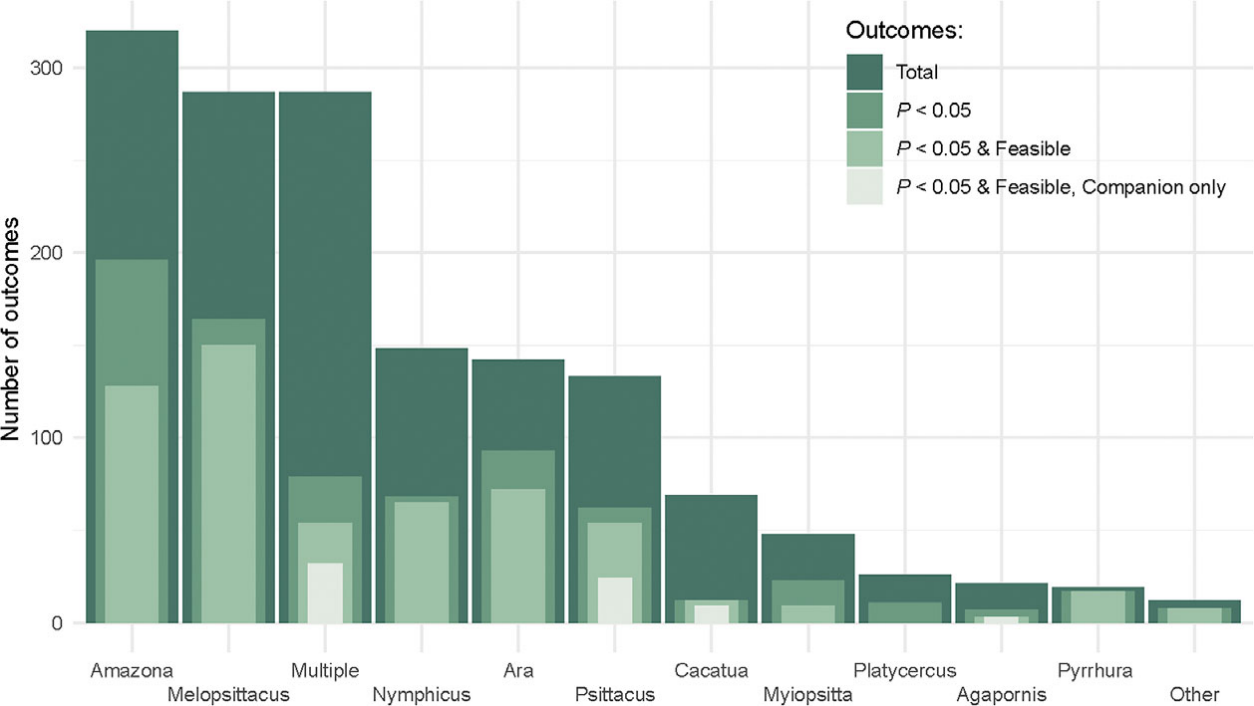
Number of welfare outcomes identified in the systematic literature search, grouped by parrot genera. For each genus, the overlapped bar plot shows, from darkest to lightest colour, the total number of welfare-related outcomes collected, the number of significant outcomes (*P* < 0.05), the number of significant outcomes that are considered feasible for evaluation by owners (i.e. not requiring any particular skill, expertise or equipment; next-to-smallest bar plot), and the number of significant and feasible outcomes that were obtained from companion parrots (smallest bar plot). The genus ‘Other’ refers to the pooled genera *Calyptorhynchus* (black cockatoos), *Guaruba* (golden conures) and *Loriculus* (hanging parrots).

Outcome measures were collected from 13 genera, of which ten belonged to the superfamily *Psittaccoidea* and three to the superfamily *Cacatuoidea. Melopsittacus* (budgerigars) and *Amazona* (Amazon parrots) were the genera with the highest number of significant and feasible outcomes (n = 150 and n = 128, respectively), followed by the *Ara* (macaws; n = 72)*, Nymphicus* (cockatiels; n = 65), and *Psittacus* (grey parrots; n = 54) genera (Figure 3, Table S8; Supplementary material). Fifty-four (9.44%) significant and feasible outcome measures were available from studies that included multiple genera (see Table S7; Supplementary material). For other genera, the number of significant and feasible outcomes collected ranged between 1 (*Guaruba;* golden conures) and 17 (*Pyrrhura;* conures). For the genus *Platycercus* (rosellas) no significant and feasible outcome measures were identified (Figure 3, Table S8; Supplementary material). A total of 68 feasible and significant outcome measures were collected from companion parrots, with 32 (47%) outcomes related to multiple genera, 24 (35.4%) to the genus *Psittacus*, nine (13.2%) to the genus *Cacatua* (cockatoos), and three (4.4%) to the genus *Agapornis* (lovebirds) (Figure 3, Table S12; Supplementary material).

### Relationship between genera and welfare dimensions

**Figure 4.**
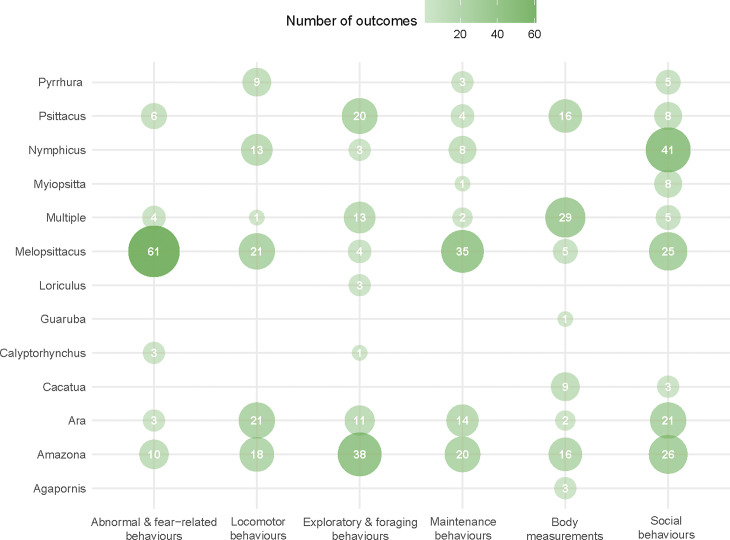
Bubble plot showing the number of significant and feasible outcome measures related to parrot welfare identified during the systematic literature search (n = 572). The numbers inside the bubbles correspond to the number of outcome measures identified for each welfare dimension (x-axis) – parrot genus (y-axis), while size and colour of the bubbles reflect the relative proportion of outcomes covered by each combination; hence, larger and darker bubbles represent a combination for which a higher number of significant and feasible outcomes were reported.

Of the welfare dimensions for which we identified significant and feasible outcomes, ‘social behaviours’ was the one investigated in the highest number of genera (nine out of 13). All other welfare dimensions covered eight genera, except the dimensions ‘abnormal and fear-related behaviour’ and ‘locomotor behaviour’ which both were covered by six genera (Figure 4). The genera-welfare dimension association with the highest number of feasible and significant outcomes was the combination *Melopsittacus* – ‘abnormal and fear-related behaviours’ (n = 61), followed by *Nymphicus* – ‘social behaviours’ (n = 41), *Amazona* – ‘exploratory and foraging behaviours’ (n = 38), *Melopsittacus* – ‘maintenance behaviours’ (n = 35), and ‘multiple genera’ – ‘body measurements’ (n = 29) (Figure 4). Three genera were covered by only one welfare dimension: *Agapornis* and *Guaruba* with ‘body measurements’, and *Loriculus* with ‘exploratory and foraging behaviours’ (Figure 4).

## Discussion

### Internal validity

The main aim of this systematic study was to identify potential welfare indicators for companion parrots by first assessing the internal validity of outcome measures from published scientific studies. We found a high risk of bias associated with the outcome measures in the scientific literature. For instance, intra- and inter-observer reliabilities and assessor blindness were almost never reported. We identified 572 outcomes measures that presented a significant *P*-value and that we classified as feasible, but these need to be thoroughly validated before being used as welfare indicators in practical assessments (European Food Safety Authority [EFSA] 2012).

### Welfare dimensions

The significant and feasible outcome measures linked to parrot welfare were well distributed across six of the eight welfare dimensions, except for physiological parameters, and disease and pathological conditions. Behaviours represented the most common type of outcomes measures, covering five out of eight welfare dimensions, and 24 out of 34 categories.

‘Social behaviour’ was the welfare dimension with the greatest variety of significant and feasible outcome measures and welfare categories. Within this dimension, we found several behaviours such as vocalisation, mate-related behaviours, aggressiveness, allopreening, or behaviours related to human-animal interactions. The latter presented a remarkable heterogeneity of outcome measures, including several outcomes linked to inappropriate handling and physical contact. Human-animal relationship plays a fundamental role in guaranteeing companion animals’ positive welfare (Rault *et al.* 2020); for this reason, these results represent a good starting point for the development of an assessment tool tailored to companion parrots. Outcome measures of the welfare category, ‘facial and body display’ were the only results retrieved that proposed the use of facial expressions (feather ruffling, blushing) as indicators of calmness and positive human-parrot interaction (Bertin *et al.* 2018, 2020, 2023) and the display of erected crests as a sign of high arousal (Lievin-Bazin *et al.* 2018). Many other parrots’ displays, such as body postures, have been interpreted as ways parrots communicate their level of arousal or signal an imminent aggressive response (Wilson 2022), however this information is not supported by experimental studies. Observing facial and body displays can be useful to assess welfare, for example, in preventing negative interactions with caretakers or other animals that live in the same environment; however, further investigations are needed to validate such indicators.

Three welfare dimensions concentrated almost half of the significant and feasible outcomes: ‘locomotor behaviours’, in which we grouped behaviours such as flying and climbing; ‘exploratory and foraging behaviours’, reflecting the way in which parrots explore new environments and novel objects and interact with enrichment; and ‘maintenance behaviours’, in which we clustered behaviours such as feeding and resting. All these behaviours are used while foraging, an important activity for wild parrots as it occupies between 40 and 70% of their daily active time (Magrath & Lill 1983; Westcott & Cockburn 1988). Therefore, indicators linked to foraging are suggested to be relevant to monitor captive parrot welfare.

For the welfare dimension ‘abnormal and fear-related behaviours’ we retrieved several outcomes that could be used to assess welfare: incessant screaming, phobic behaviours, and many types of stereotypies, such as locomotor (e.g. route trace, pacing), oral (e.g. wire chewing) and whole body (e.g. rocking, bobbing) stereotypies. Feather-damaging behaviour, a common abnormal behaviour in companion parrots with a wide range of underlying causes, including medical issues, socio-environmental factors and neurochemical changes (van Zeeland *et al.* 2009), affects between 11.7 and 25.4% of the overall parrot population, according to surveys conducted in different countries (Kinkaid *et al.* 2013; Costa *et al.* 2016; Ebisawa *et al.* 2021; Mellor *et al.* 2021; Mahdavi *et al.* 2023). Grey parrots (*Psittacus erithacus*) and cockatoos are reported to be predisposed to develop this problem (Seibert 2006a; Kinkaid *et al.* 2013), with surveys indicating a prevalence between 22.5 and 39.4% in grey parrots (Jayson *et al.* 2014; Costa *et al.* 2016; Ebisawa *et al.* 2021; Mahdavi *et al.* 2023) and between 30.6 and 42.4% in cockatoos (Kinkaid *et al.* 2013; Jayson *et al.* 2014; Ebisawa *et al.* 2021). Nevertheless, other species, including lovebirds, pacific parrotlets (*Forpus coelestis*), red-shouldered macaws (*Diopsittaca nobilis*), conures (*Aratinga* spp, *Pyrrhura* spp), and eclectus parrots (*Eclectus roratus*) have also emerged as species that are seemingly prone to develop this behaviour (Kinkaid *et al.* 2013; Costa *et al.* 2016; Ebisawa *et al.* 2021). Due to its high prevalence and association with several medical problems (van Zeeland *et al.* 2009), feather-damaging behaviour has been the subject of many studies. However, feather-damaging behaviour is difficult to observe directly, which may explain why we retrieved only one study where authors recorded duration and frequency of this self-injuring behaviour (Seibert *et al.* 2004). Stereotypies and feather-damaging behaviour, however, do not always reflect the current welfare state of the subject as they may also manifest themselves as “*behavioural scars*” and can remain even after the stressful stimulus or situation that triggered them is no longer present (Mason 1991). For this reason, they should be included in a parrot welfare assessment scheme but accompanied by the corresponding risk factors identified, such as being hand-reared or single-housed, lack of enrichment, or living in a small cage.

‘Body measurements’ was the only welfare dimension to include significant and feasible outcome measures that were not behaviours and consisted of two categories. One was ‘indirect measures of feather-damaging behaviours’ and included the outcomes ‘presence of feather damage (yes/no)’ and plumage scores. These two outcomes can be used to detect the presence of feather-damaging behaviour. Additionally, plumage scores allow caretakers to monitor improvement or deterioration of plumage condition over time. Moreover, previous studies showed good to excellent agreements within and between observers for this type of measurement (van Zeeland *et al.* 2013b; Mellor *et al.* 2023). It is important to highlight that damage to the plumage is not specific to behavioural disorders, as it can also be caused by other factors that are still relevant to parrot welfare such as malnutrition, virus infections, parasitic infestation or inappropriate husbandry or management (e.g. small or overcrowded cages) (van Zeeland & Schoemaker 2014). Due to their high feasibility and their well-established link to the welfare of captive parrots, ‘indirect measures of feather-damaging behaviours’ can be considered the most promising welfare indicators among all types of outcomes collected. The second welfare category in the dimension ‘body measurements’ was ‘body condition’, which contained measures indirectly linked to body fat composition, such as body weight, chest girth, and body mass. Despite the lack of data on the prevalence of obesity in the companion parrot population, this condition is regarded as a common welfare problem in companion parrots, especially in budgerigars (*Melopsittacus undulatus*), cockatiels (*Nymphicus hollandicus*), Amazon parrots and galahs (*Eolophus roseicapilla*) (Speer *et al.* 2016; Chitty 2023); its emergence is believed to be linked to a combination of unbalanced diets, selective eating, and lack of exercise (Harrison *et al.* 2006; Chitty 2023). Several studies included in this systematic review tested the effect of an unbalanced diet on parrots’ body condition; however, most of the results were not significant. Caloric deficit and surplus are responsible for changes in body fat composition in most animal species and, arguably, this also likely applies to parrots. However, some studies found that body mass decreases in association with regular physical activity (Schnegg *et al.* 2007; Gustavsen *et al.* 2016) and increases with a prolonged exposure to artificial light at night (Malek *et al.* 2020). Changes in body composition and the factors influencing these changes appear to be understudied, yet they could be highly relevant to the welfare of companion parrots.

We could not retrieve any feasible outcomes for the welfare dimension ‘physiological parameters’, mostly due to these requiring specialised techniques or equipment for collection or analysis. However, we identified several risk factors that can be associated with changes in these physiological parameters. Advancements in new technologies or methodologies could enhance welfare assessments by enabling caregivers to collect non-invasive physiological measurements. However, until such advancements are achieved, and widely available, veterinarian input may be necessary to obtain a more complete picture of parrot welfare. Husbandry and management conditions were recurrent risk factors that influenced parrots’ physiology, especially stress-related parameters. For the welfare dimension ‘diseases and pathologic conditions’, which also lacked feasible outcome measures, risk factors mostly included demographic characteristics like parrot age, sex, or species. None of the studies retrieved looked for physical measurements as a clinical sign for existing disease or pathology. This finding was unexpected considering that these types of measurements are heavily influenced by health problems; for instance, upper beak and nail overgrowth, increased weight, and changes in feather colour and quality are anecdotally reported as signs indicative of (fatty) liver disease (Grunkemeyer 2010). Some of these parameters, commonly used by veterinarians based on expert knowledge or experience, were not reflected in the scientific literature or may have been missed in our search as it is difficult to comprehensively capture all possible health problems. Further experimental validation of some of these commonly used clinical diagnostic signs would be valuable.

### Most relevant risk factors for companion parrots

Among all risk factors associated with poor welfare, four emerged as especially important for the welfare of companion parrots. As suggested by several authors, captive parrots need to be mentally stimulated with different types of enrichment in order to prevent boredom, frustration, and other conditions associated with poor welfare (Livingstone 2018; Seibert 2020). In support of this, we found that a lack of physical and foraging enrichment was the most recurrent risk factor and was associated with changes in maintenance, locomotor, exploratory and social behaviours, and with expression of stereotypies and feather-damaging behaviour. A recent study on grey parrots, not included in our results, demonstrated that combining two different enrichment devices stimulated both the appetitive and consummatory phases of foraging behaviour, resulting in increased daily foraging time (Beekmans *et al.* 2023); hence not just the provision but also the design of enrichment devices is important. Moreover, other forms of enrichment that have been less investigated (e.g. cognitive and auditory) warrant research.

Social deprivation and social isolation also appeared to be recurrent risk factors in our results and were associated with outcome measures belonging to seven out of the eight welfare dimensions. Parrots are highly social species (Seibert 2006b) but are often housed alone as companion animal, a living condition that we found being linked to poor welfare. A recent study showed that parrots living without other parrots were more likely to show problematic behaviours such as biting humans and stealing human food, and parrots left alone for more than 6 h daily tended to be more prone to show feather-damaging behaviour (Tygesen & Forkman 2023).

Personality also influences how parrots interact with their environment and cope with challenging situations. Several studies included in this systematic review showed that specific personality traits or coping styles were linked to the emergence of feather-damaging behaviour (van Zeeland *et al.* 2013a), to the exhibition of attention bias (Cussen & Mench 2014), to the time spent feeding and interacting with the enrichment (Ramos *et al.* 2021), and to fearful responses towards humans (Franzone *et al.* 2022). Assessing personality may be an effective strategy for improving the welfare of captive animals (Wilson *et al.* 2019) although the best methods of determining personality remain debated (Richter & Hintze 2019).

Rearing methods also emerged as a crucial risk (developmental) factor that may impact parrots’ quality of life and welfare. Neonatal handling of parent-reared chicks can result in reduced aggressiveness and fear-related and feather-damaging behaviours in later life (Collette *et al.* 2000; Fox & Millam 2004), whereas hand-rearing has been linked to these problematic behaviours (Schmid *et al.* 2006; Costa *et al.* 2016; Ebisawa *et al.* 2022), and to issues related to sexual imprinting, resulting in social and sexual preference for humans and impaired social bonds with conspecifics (Fox 2006). However, given that hand-rearing might induce irreversible changes, the results observed from the studies should be used with informative and preventive purpose, as these cannot be changed after weaning.

Overall, our data point to a lack of enrichment, social isolation, personality, and rearing method as important aspects for companion parrots that should be taken into account in parrot welfare assessment.

### External validity

The second aim of this systematic review was to assess the external validity of the outcomes by establishing from which species data were obtained and in which settings the studied subjects lived. We found two important factors that potentially compromise external validity of the outcomes collected: the presence of a strong taxonomic bias, and an overrepresentation of results from studies of parrots kept in laboratories. The term ‘taxonomic bias’ refers to differences in our knowledge of certain species and the degree to which they are the subject of scientific investigation across a wide variety of biological fields (Troudet *et al.* 2017). The fact that amazon parrots, budgerigars, and cockatiels received more research attention compared to other species such as cockatoos, monk parakeets (*Myiopsitta monachus*), or lovebirds clearly demonstrates a bias in the scientific literature. Several factors might have contributed to this discrepancy. For instance, parrots like budgerigars and cockatiels are easily found, possess lower economic value, are easy to handle and tend to have a short generation interval as they become sexually mature before one year (Kavanau 1987). All these characteristics, typical of commonly used animal models, make these species good candidates to conduct scientific studies under laboratory conditions. Although lovebirds possess similar characteristics, only one study on this genus met our inclusion criteria. Amazon parrots do not possess any of these characteristics, yet were the most studied species, with the second highest number of significant and feasible outcome measures. This is explained not by the widespread study of these species, but rather by the large amount of information gathered from one laboratory at the University of California, Davis, which published several studies on this taxon. We retrieved scarce data for black cockatoos (*Calyptorhynchus* spp), golden conures (*Guaruba guarouba*), and hanging parrots (*Loriculus* spp), but this is not surprising as these genera are mostly kept in zoos and breeding facilities and are rarely used in laboratory settings or kept as companion parrots. Nonetheless, no studies could be found for certain species that are commonly kept in captivity, including ring-necked parrots (*Psittacula* spp), caiques (*Pionites* spp), galahs and eclectus.

Studies conducted on multiple genera included these taxa along with many others, however the information obtained from these studies should be evaluated carefully before being used for individual species assessments. Some taxonomic groups have in fact specific needs and show different sensitivities when exposed to similar environmental stimuli, making generalisation of findings to other genera sometimes difficult or irrelevant. It should be emphasised that data extrapolation should be performed with caution even within the same genus, as for example *Amazona*, *Ara* and *Cacatua* each contain several species adapted to different natural habitats and showing distinct behaviours within the same genus (Parr & Juniper 2010). As such, extrapolating findings to other species, even within the same genus, may be improper and counterproductive, emphasising the need for further research to bridge these knowledge gaps for understudied species.

Our results show that certain welfare dimensions and categories were investigated only in a limited number of genera. For instance, we identified outcomes such as abnormal, locomotor, exploratory, and foraging behaviours for Amazon parrots, macaws, and budgerigars, but these parameters were not described in lovebirds, cockatoos, and monk parakeets. It is important to underline that the absence of information related to some taxa in our findings was not necessarily due to a lack of scientific studies but rather to a lack of significant results. For instance, we found that the provision of physical enrichment was linked to changes of preening in Amazon parrots, macaws and conures (Van Hoek & King 1997; Cussen & Mench 2015; Reimer *et al.* 2016; Almeida *et al.* 2018), but not in cockatiels (Carvalho *et al.* 2017; Stevens et al. 2021). In fact, none of the results obtained from this genus presented a significant *P*-value. This could mean that cockatiels, unlike other parrot species, do not show changes in preening behaviour in these situations or, alternatively, that experimental set-ups and methods applied in the studies were not able to detect such changes.

As mentioned above, more than half of the outcomes were found from studies conducted in laboratory settings. Laboratories are highly controlled environments where daily routines related to animal care and testing are highly standardised. Such settings theoretically ensure results are more reproducible, but only in the case that characteristics are similar to those from which original data were extrapolated. This condition, defined as “*standardization fallacy*”, can lead to a decrease of external validity (Würbel 2000). In our case, external validity is important as parrot welfare indicators should ideally be applicable across parrots of different species and existing in various living conditions. Although zoos, shelters, rehabilitation centres, and breeding facilities offer settings that are not as standardised as in laboratories, they still differ greatly from domestic environments, which could be a cause for concern when extrapolating these findings to companion parrots.

### Companion parrots

We found limited information related to companion parrots in terms of the number and variety of outcomes measures. In fact, the number of feasible and significant outcomes retrieved from companion parrots was less than 5% of the total outcome measures collected, and 75% of those were related to feather-damaging behaviour. Moreover, half of these outcomes for companion parrots were obtained from only three genera: *Psittacus*, *Cacatua* and *Agapornis*, with *Psittacus* being the only genus that covered the entire breadth of welfare categories, and outcomes for the other two genera being restricted to those related to feather-damaging behaviour. While these three genera are among the most commonly kept companion parrot species, several other species, such as macaws, Amazon parrots, conures, caiques, parrotlets, budgerigars, and other parakeets are also popular as companions, for which no significant and feasible outcomes were identified. An additional problematic aspect is that almost all outcome measures for companion parrots were obtained through questionnaires. Prospective, case-control studies might be challenging to perform with companion parrots under experimental circumstances as parrots may be harder to recruit, and possibly be less adaptable to new environments and/or unfamiliar humans compared to dogs and cats, which could lead to altered behavioural responses. Questionnaires may represent the best way to study this specific cohort and gather large amounts of data while preventing potential discomfort in the studied parrots. However, questionnaires are also highly sensitive to bias (Choi & Pak 2005), and therefore results obtained using this method should be applied cautiously and possibly require complementary experimental testing. Overall, these results emphasise the need for more research on companion parrots, especially on species and welfare dimensions that have been underrepresented in this cohort. Some welfare indicators from other companion animal species could also be relevant and applicable to parrots, but we decided to focus on the literature on parrots because it offers greater external validity, and considering the already large number of parrot taxa and species with heterogenous needs.

## Study limitations

This systematic review presents some limitations. The first is a lack of terms used to create the search queries, especially those related to medical conditions. This would certainly have allowed to increase the final number of studies retrieved; however, due to the high number of diseases and pathologic conditions, we decided to select terms related to specific medical conditions. The second important limiting factor was the fact that data collection was carried out by only one person (AP), which might have led to missing information or to systematic errors that reduced the accuracy of the results presented. The third and final limitation is related to the low internal validity and the selection of significant outcomes. The *P*-value significance is a necessary condition to reflect the sensitivity of an outcome measure as a potential welfare indicator. However, when studies present high risk of bias, the *P*-value can be influenced by confounding factors, leading to false negative or false positive results (Ioannidis 2005). Further studies should include the effect size as a parameter to assess internal validity with higher confidence, but this was not possible in this study based on the heterogeneity of the outcomes collected and the information provided by authors (e.g. lack of reporting of effect size).

## Animal welfare implications and conclusion

The purpose of this systematic review was to identify valid and feasible welfare indicators for companion parrots. Despite the large amount of information collected, we could only identify plumage condition as a previously validated and feasible parrot welfare indicator. In fact, the lack of information contained in the publications made it difficult to assess the internal validity of the outcome measures. Moreover, given the lack of experimental studies focused on parrot health, future research should aim to validate physical parameters commonly used by veterinarians as clinical indicators of disease, metabolic disorders, or nutritional deficiencies. We also noticed potentially low external validity due to taxonomic bias and an overrepresentation of studies on parrots kept in laboratories. These challenges to ascertain validity prevented us from establishing a definitive list of reliable and useful welfare indicators for companion parrots. Nevertheless, this systematic review helps to summarise the current state of scientific knowledge on aspects relevant to parrot welfare (a dataset containing all data collected from the studies is available as Supplementary material for further reference) and identifies a list of potential welfare indicators for use in parrots. Future directions should focus on validating the identified welfare indicators. This is essential to create a comprehensive and reliable welfare assessment that accurately reflects the overall well-being of companion parrots.

## Supporting information

Piseddu et al. supplementary material 1 Piseddu et al. supplementary material

Piseddu et al. supplementary material 2 Piseddu et al. supplementary material

## Supplementary material

The supplementary material for this article can be found at http://doi.org/10.1017/awf.2024.61.
