# Supplementary material for: What we (don’t) know about parrot welfare: Finding welfare indicators through a systematic literature review
Source: Anim Welf. 2024 Dec 5;33:e57. doi: 10.1017/awf.2024.61 (PMC11655281; doi:10.1017/awf.2024.61)
Supplement: Piseddu et al. supplementary material 2 — Piseddu et al. supplementary material [file S0962728624000617sup002.pdf]

Supplementary material

What We (Don't) Know about Parrot Welfare: finding welfare indicators through a systematic literature review

Andrea Piseddu[1*], Yvonne R. A. van Zeeland[2] and Jean-Loup Rault[1]

[1]Centre for Animal Nutrition and Welfare, University of Veterinary Medicine Vienna, Veterinaerplatz 1, 1210 Vienna, Austria
[2]Division of Zoological Medicine, Department of Clinical Sciences, Faculty of Veterinary Medicine, Utrecht University, Yalelaan 108, 3584 CM Utrecht, The Netherlands
*Author for correspondance : andrea.piseddu@vetmeduni.ac.at

Materials and methods

Search Query creation

A search query was created using the terms reported in Table S1 and additional terms included in the thesaurus of the databases. Filters related to language, publication type and terms to exclude from the results of the systematic search (e.g. "wild birds") were also included in the search queries.

Table S1. Terms used to create the search queries for the systematic literature search to identify valid and feasible outcome measures to assess the welfare of companion parrots.

Search components	Terms
**Population**	parrot, psittacines, *Psittacids*, cockatoo, macaw, parakeet, budgerigar, cockatiel, *Ara*, *Cacatua*, *Psittacus*, african grey parrot, "grey parrot", amazon parrot, lovebirds, *Poicephalus, Agapornis*, *Psittaccula*, *Eclectus*, conure, caique.
**Intervention**	enrichment, environmental enrichment, social enrichment, nutritional enrichment, physical enrichment, sensory enrichment, deprivation, diet, nutrition, malnutrition, play, toy, puzzle, play activity, foraging, foraging toy, foraging activity, social activity, activity, stimulation, social bond, social relation, cognitive stimulation, attachment, hand rearing, parent rearing, co-parenting
**Outcomes**	feather picking, feather plucking, feather damaging, self-damaging, self-mutilation, pododermatitis, atherosclerosis, metabolic bone disease, body condition, obesity, nutritional deficiency, injuries, disease, stereotypes, stereotypical behaviours, abnormal behaviours, behavioural disorders, destructive behaviour, egg laying, reproduction, fertility, biting, screaming, excessive vocalisation, natural behaviours, aggressive, emotion, emotional state, positive state, negative state, stress, distress, hormones, corticosterone, lifespan, life expectancy, longevity, aging

For each database, we applied filters in order to exclude irrelevant studies and optimise the results of the systematic search. These filters included the language restriction (only English), the exclusion of studies focused on wild parrots and reviews or case studies. From a preliminary systematic search, we identified irrelevant studies (e.g. studies focused on bacteria, plants, mammals etc), and therefore added online filters offered by the databases. This included filters related to the topic, authors name (e.g. Parrot *et al*) and studies category (e.g. Fishery, Marine Freshwater Biology, Sport Sciences etc). In addition, on PubMed we applied relevant MeSH terms and the filter "Title/Abstract", on CAB direct relevant organism descriptors and topic terms.

Systematic Search Queries

Below are the search queries used on the 3 databases.

PubMed

((((((((((((((((((((((((((((Parrot[Title/Abstract]) OR (Psittacines[Title/Abstract])) OR (Psittacids[Title/Abstract])) OR (Psittacidae[Title/Abstract])) OR (Psittacinae[Title/Abstract])) OR (Cacatuidae[Title/Abstract])) OR (Cockatoo[Title/Abstract])) OR (Macaw[Title/Abstract])) OR (Parakeet[Title/Abstract])) OR (Budgerigar[Title/Abstract])) OR (Cockatiel[Title/Abstract])) OR) OR (Cacatua[Title/Abstract])) OR (Psittacus[Title/Abstract])) OR (Grey parrot[Title/Abstract])) OR (Amazona[Title/Abstract])) OR (lovebird[Title/Abstract])) OR (Agapornis[Title/Abstract]))) OR (Psittacula[Title/Abstract])) OR (Conure[Title/Abstract])) OR (Caique[Title/Abstract])) OR (Ecletus[Title/Abstract])*) OR (Poichephalus[Title/Abstract])*) OR ("Parrots"[MAJR])) OR ("Cockatoos"[MAJR])) AND (("Feather picking") OR ("feather plucking")) OR ("feather damaging")) OR ("feather destruction")) OR (self-

damaging)) OR (self-mutilation)) OR (automutilation)) OR (pododermatitis)) OR (atherosclerosis)) OR ("metabolic bone desease")*) OR ("Body condition")) OR (obesity)) OR (injury)) OR (disease)) OR ("nutritional deficiency")) OR (Stereotypes)) OR ("stereotypical behaviour")) OR ("abnormal behaviour")) OR ("repetitive behaviour")*) OR ("behavioural disorder")) OR ("destructive behaviour")) OR ("natural behaviour")) OR ("egg laying")) OR (reproduction)) OR (fertility)) OR (aggression)) OR (emotion)) OR ("emotional state")) OR (biting)) OR (screaming)) OR ("excessive vocalization")) OR ("positive state")) OR ("negative state")) OR (Stress)) OR (distress)) OR (hormones)) OR (corticosterone)) OR (Lifespan)) OR ("life expectancy")) OR (longevity)) OR (aging)) OR ("Bird Diseases/diagnosis"[MeSH])) OR ("Cognition"[MeSH])) OR ("Emotions"[MAJR])) OR ("Parrots/physiology"[MAJR])) OR ("Vocalization, Animal"[MAJR])) OR ("Feathers/injuries"[MAJR])) OR ("Corticosterone/blood"[MeSH])) OR ("Diagnosis, Differential"[MeSH])) OR ("Aggression/physiology"[MAJR])) OR ("Stereotyped Behavior"[MAJR])) OR ("Impulsive Behavior"[MeSH])) OR ("Behavior, Animal"[MeSH])) OR ("Feeding Behavior/physiology"[MAJR])) OR ("Amazona/metabolism"[MAJR])) OR ("Amazona/physiology"[MAJR])) OR ("Amazona/growth and development"[MAJR])) OR ("Animal Feed/analysis"[MAJR]))) OR ((((((((((((((((((((((((Parrot[Title/Abstract]) OR (Psittacines[Title/Abstract])) OR (Psittacids[Title/Abstract])) OR (Psittacidae[Title/Abstract])) OR (Psittacinae[Title/Abstract])) OR (Cacatuidae[Title/Abstract])) OR (Cockatoo[Title/Abstract])) OR (Macaw[Title/Abstract])) OR (Parakeet[Title/Abstract])) OR (Budgerigar[Title/Abstract])) OR (Cockatiel[Title/Abstract])) OR) OR (Cacatua[Title/Abstract])) OR (Psittacus[Title/Abstract])) OR (Grey parrot[Title/Abstract])) OR (Amazona[Title/Abstract])) OR (lovebird[Title/Abstract])) OR (Agapornis[Title/Abstract]))) OR (Psittacula[Title/Abstract])) OR (Conure[Title/Abstract])) OR (Caique[Title/Abstract])) OR (Ecletus[Title/Abstract])*) OR (Poichephalus[Title/Abstract])*) OR ("Parrots"[MAJR])) OR ("Cockatoos"[MAJR])) AND ((((((((((((((((((((((((((((((Enrichment) OR ("Environmental Enrichment")) OR ("Social Enrichment")) OR ("Nutritional Enrichment")) OR ("Physical Enrichment")) OR ("sensory enrichment")) OR (Deprivation)) OR (Diet)) OR (Nutrition)) OR (Malnutrition)) OR (Play)) OR (Toy)) OR (Puzzle)) OR ("Play activity")) OR (Foraging)) OR ("Foraging toy")) OR ("foraging activity")) OR ("social activity")) OR (activity)) OR (stimulation)) OR ("social bond")) OR ("social relation")) OR ("cognitive stimulation")) OR (attachment)) OR ("Hand rearing")) OR ("parent rearing")) OR (co-parenting)) OR ("Behavior, Animal/drug effects"[MeSH])) OR ("Animal Nutritional Physiological Phenomena/physiology"[MeSH])) OR ("Acoustic Stimulation"[MeSH])) OR ("Social Isolation/psychology"[MAJR])) OR ("Play and Playthings"[MAJR]))) AND (english[Filter] OR french[Filter] OR italian[Filter])) NOT (Review[Publication Type])) NOT (Case Reports[Publication Type])) NOT ("wild birds") AND (english[Filter] OR french[Filter] OR italian[Filter])

*Misspelled terms

CAB direct

((((((((up:("Cacatuidae" or "Psittacidae")) OR ((up:("Psittaciformes" or "Psittacus")) OR (((((((Parrot) OR (Psittacines) OR (Psittacids) OR (Psittacidae) OR (Psittacinae) OR (Cacatuidae) OR (Cockatoo) OR (Macaw) OR (Parakeet) OR (Budgerigar) OR (Cockatiel) OR (Ara) OR (Cacatua) OR (Psittacus) OR (Grey parrot) OR (lovebird) OR (Agapornis) OR (Psittacula) OR (Conure) OR (Caique) OR (Ecletus)* OR (Poichephalus)*))) OR (od:("Ara" or "Amazona" or "Cacatua" or "Eclectus roratus" or "Nymphicus hollandicus" or "Psittacula" or "Myiopsitta monachus" or "Cacatuidae" or "Nymphicus" or "Pionites" or "Psittacus" or "Eclectus" or "Aratinga")))) OR (od:("Cacatua" or "Agapornis" or "budgerigars"))))) AND ((de:("behaviour problems")) OR (((id:("chewing")) OR (((((((("Feather picking") OR ("feather plucking") OR ("feather damaging") OR ("feather destruction") OR (self-damaging) OR (self-mutilation) OR ("automutilation") OR (pododermatitis) OR (atherosclerosis) OR ("Body condition") OR (obesity) OR (injury) OR (disease) OR ("nutritional deficiency") OR (Stereotypes) OR ("stereotypical behaviour") OR ("abnormal behaviour") OR ("repetetive behaviour")* OR ("behavioural disorder") OR ("destructive behaviour") OR ("natural behaviour") OR ("egg laying") OR (reproduction) OR (fertility) OR (aggression) OR (emotion) OR ("emotional state") OR (biting) OR (screaming) OR ("excessive vocalization") OR ("positive state") OR ("negative state") OR (Stress) OR (distress) OR (hormones) OR (corticosterone) OR (Lifespan) OR ("life expectancy") OR (longevity) OR (aging))) OR (de:("corticosterone" or "behaviour" or "feather pecking" or "animal behaviour" or "egg production")))) OR (id:("pterotillomania" or "cage birds" or "feather damaging behaviour" or "captive animals")))) OR (id:("deviant behaviour" or "abnormal behavior")))) OR (id:("chewing"))))) OR (((((((Enrichment) OR ("Environmental Enrichment") OR ("Social Enrichment") OR ("Nutritional Enrichment") OR ("Physical Enrichment") OR ("sensory enrichment") OR (Deprivation) OR (Diet) OR (Nutrition) OR (Malnutrition) OR (Play) OR (Toy) OR (Puzzle) OR (Play activity) OR (Foraging) OR (Foraging toy) OR (foraging activity) OR (social) OR (activity) OR (stimulation) OR (social bond) OR (social relation) OR (cognitive stimulation) OR (attachment) OR (Hand rearing) OR (parent rearing) OR (co-parenting))) OR (de:("enrichment" and "physical activity" and "quality of life" and "ornamental birds" and "toys" and "animal welfare" and "foraging")))) OR (de:("exercise" or "animal nutrition" or "diets" or "ontogeny"))) AND ((up:("Cacatuidae" or "Psittacidae")) OR ((up:("Psittaciformes" or "Psittacus")) OR (((((((Parrot) OR (Psittacines) OR (Psittacids) OR (Psittacidae) OR (Psittacinae) OR (Cacatuidae) OR (Cockatoo) OR (Macaw) OR (Parakeet) OR (Budgerigar) OR (Cockatiel) OR (Ara) OR (Cacatua) OR (Psittacus) OR (Grey parrot) OR (Amazona) OR (lovebird) OR (Agapornis) OR (Psittacula) OR (Conure) OR (Caique) OR (Ecletus)* OR (Poichephalus)*))) OR (od:("Ara" or "Amazona" or "Cacatua" or "Eclectus roratus" or "Nymphicus hollandicus" or "Psittacula" or "Myiopsitta monachus" or "Cacatuidae" or "Nymphicus" or "Pionites" or "Psittacus" or "Eclectus" or "Aratinga")))) OR (od:("Cacatua" or "Agapornis" or "budgerigars")))))) NOT (Review)) NOT (de:("wild birds"))) NOT ("Case Study")) NOT (de:("DNA cloning" or "interferon-gamma"))) NOT (de:("guided tours" or "cultural tourism" or "ecotourism"))) AND (((NOT (organism-descriptor:(("Protozoa" OR "mice" OR "fowls" OR "fishes" OR "plants")))) (NOT (topic:(("aquatic organisms" OR "aquatic species" OR "aquatic animals" OR "case reports" OR "forests" OR "bacterium")))) (NOT (broader-term:(("mammals" OR "Spermatophyta" OR "angiosperms" OR "plants"))))))) AND (((topic:(("cage birds" OR "aviary birds" OR "behavior" OR "behaviour" OR "animal behavior" OR "pet animals" OR "pets" OR "animal behaviour"))) (NOT (topic:(("Viral diseases" OR "viral infections")))) (language:(("English" OR "French" OR "Italian")))))

*Misspelled terms

Web of Science

Web of Science did not allow to create a search query containing more than 100 terms, for this reason terms related to the intervention and the outcomes were reduced.

((((((((((((((((((((ALL=(Parrot)) OR ALL=(Psittacines)) OR ALL=(Psittacids)) OR ALL=(Psittacidae)) OR ALL=(Psittacinae)) OR ALL=(Cacatuidae)) OR ALL=(Cockatoo)) OR ALL=(Macaw)) OR ALL=(Parakeet)) OR ALL=(Budgerigar)) OR ALL=(Cockatiel)) OR ALL=(Ara)) OR ALL=(Cacatua)) OR ALL=(Psittacus)) OR ALL=("Grey parrot")) OR ALL=(Amazona)) OR ALL=(lovebird)) OR ALL=(Agapornis)) OR ALL=(Psittacula)) OR ALL=(Conure)) OR ALL=(Caique)) OR ALL=(Ecletus)*) OR ALL=(Poichephalus)* AND (((((((((((((((((ALL=(Enrichment)) OR ALL=("sensory enrichment")) OR ALL=(Deprivation)) OR ALL=(Diet)) OR ALL=(Nutrition)) OR ALL=(Malnutrition)) OR ALL=(Play)) OR ALL=(Toy)) OR ALL=(Puzzle)) OR ALL=(Foraging)) OR ALL=("social")) OR ALL=(activity)) OR ALL=(stimulation)) OR ALL=("social bond")) OR ALL=("cognitive stimulation")) OR ALL=("attachment")) OR ALL=(rearing)) OR ALL=(co-parenting) AND ((((((((((((((((((((((((((((((((ALL=(Feather)) OR ALL=(damage)) OR ALL=(destruction)) OR ALL=(mutilation)) OR ALL=(pododermatitis)) OR ALL=(atherosclerosis)) OR ALL=("metabolic bone desease")*) OR ALL=("Body condition")) OR ALL=(obesity)) OR ALL=(injury)) OR ALL=(disease)) OR ALL=("nutritional deficiency")) OR ALL=(Stereotypes)) OR ALL=("stereotypical behaviour")) OR ALL=("abnormal behaviour")) OR ALL=("repetitive behaviour")*) OR ALL=("behavioural disorder")) OR ALL=("natural behaviour")) OR ALL=("egg laying")) OR ALL=("reproduction")) OR ALL=(fertility)) OR ALL=(aggression)) OR ALL=("emotional state")) OR ALL=(biting)) OR ALL=(screaming)) OR ALL=("excessive vocalization")) OR ALL=("positive state")) OR ALL=("negative state")) OR ALL=(Stress)) OR ALL=(distress)) OR ALL=(hormones)) OR ALL=(corticosterone)) OR ALL=(Lifespan)) OR ALL=("life expectancy")) OR ALL=(longevity)) OR ALL=(aging)

*Misspelled terms

Inclusion and Exclusion Criteria

To select articles that fit with the research questions, we applied the eligibility criteria described in Table S2.

Table S2. Inclusion and exclusion criteria used to select relevant articles identified during the systematic literature review for possible welfare parameters useful to assess parrot welfare through an initial title/abstract screening and subsequent full-text evaluation.

**Population**	Inclusion criteria	Species: all species belonging to the order Psittaciformes
		Parrots living as companion animals or laboratory animals, in zoos, shelters or breeding centres
		Demographic factors: all ages, both sexes
	Exclusion criteria	Wild parrots
**Intervention**	Inclusion criteria	Enrichment: enriched parrot vs not enriched (between or within subject designs)<br>Social enrichment (e.g. intra-interspecific interactions, hand-raised vs parent raise), physical enrichment (e.g. foraging toys, changes in the aviary/cage/room), cognitive enrichment (problem solving activities, training etc.), nutritional enrichment (vegetable and/or fruits as well as seeds), etc.
		Vet Treatments: treated vs not treated
		Examination: In vivo, Post mortem
		Diet manipulation: increased/decreased levels of cholesterol/fibres/fat etc.
		Questionnaires
		Behavioural tests: novel object, open field, flight training, etc.
		Personality tests: only when result are related to the outcomes described below
**Outcomes measures**	Inclusion criteria	Problematic behaviours: aggressiveness (e.g. bites), fear-related behaviour (avoidance behaviour), abnormal behaviours (self-mutilation, feather plucking, stereotypies, incessant screaming).
		Activity level: resting, exploratory behaviour, foraging behaviour
		Social behaviours: play, allopreening, interspecific interactions, human interactions
		Vocal behaviours
		"Body language": feather ruffling, body posture
		Health measures: body weight, plumage quality, presence of diseases, mortality
		Behaviour related to basic needs: eating, drinking, resting
		Chick mortality only when related to aggressive interactions
	Exclusion criteria	Reproductive parameters: egg hatchability, num. of eggs laid
		Chicks-related: development parameters and nutritional requirements
**General**	Inclusion criteria	Only English
		Publication date restriction: none
	Exclusion criteria	Methodological details: studies that lacked statistical analysis
		Publication type: reviews, conference abstracts, book chapters
		Irretrievable studies
		Study design: case studies, studies with less than 5 subjects

Full-text screening

Two different reviewers (AP and J-LR) screened the 140 articles. The reviewers, using the exclusion and the exclusion criteria, independently select the papers that considered eligible. After the full-text screening, the reviewers compared their two lists of eligible studies and decide which ones to exclude or include. In case of disagreement between the reviewers a study was considered not eligible.

Data collection

Descriptions of the validity measures

Table S2. Definitions of the validity parameters used to assess the risk of bias of possible outcome measures related to parrot welfare as identified during the systematic literature review.

Validity parameters	Description	**Levels**
**Inter-observer reliability**	Were the outcomes tested for inter-reliability?	**Yes**: authors reported in the main text that the outcome was tested for inter-observer reliability<br>**No**: authors reported in the main text that the outcome was not tested for inter-observer reliability<br>**Not specified**: authors did not report the information in the main text<br>**Not Possible**: the experimental set up did not allow to test for inter-observer reliability
**Intra-observer reliability**	Were the outcomes tested for intra-reliability?	**Yes**: authors reported in the main text that the outcome was tested for intra-observer reliability<br>**No**: authors reported in the main text that the outcome was not tested for intra-observer reliability<br>**Not specified**: authors did not report the information in the main text<br>**Not Possible**: the experimental set up did not allow to test for intra-observer reliability
**Random group assignment**	Were the subjects randomly assigned to experimental and control groups?	**Yes**: authors reported random group assignments of the subjects in the main text<br>**No**: authors reported in the main text that the subjects were not randomly assigned to groups<br>**Not specified**: authors did not report the information in the main text<br>**Not Possible**: the experimental set up did not allow to randomly assign subjects to groups
**Condition balancing**	Were conditions balanced between subjects and/or groups?	**Yes**: authors reported in the main text that experimental conditions were balanced between subjects and/or groups<br>**No**: authors reported in the main text that experimental conditions were not balanced between subjects and/or groups<br>**Not specified**: authors did not report the information in the main text<br>**Not Possible**: the experimental set up did not allow to balance conditions between subjects and/or groups
**Observer blindness**	Was the outcome assessor blinded during data collection?	**Yes**: authors reported in the main text that the assessor was blinded<br>**No**: authors reported in the main text that the assessor was not blinded<br>**Not specified**: authors did not report the information in the main text<br>**Not Possible**: the experimental set up did not allow to blind the assessor

Welfare dimensions and outcome categories

Table S3. Welfare dimensions and corresponding outcome categories which were used to group the outcome measures related to parrot welfare as identified during the systematic literature review.

Welfare Dimensions	Outcome Categories
**Body measurements**	Indirect measures of feather-damaging behaviours, body condition, feather colour
**Physiological parameters**	Stress-related, metabolic, vitamin D-related, lipid-related, immune system-related, body temperatures, others
**Abnormal and fear-related behaviour**	Feather-damaging behaviours, fear-related, stereotypies, incessant screaming
**Maintenance behaviour**	Feeding, drinking, resting, self-care
**Locomotor behaviour**	Fly, position occupied in the cage, locomotion, inactivity
**Exploratory and foraging behaviour**	Cognitive stimulation, enrichment interaction, foraging, environment/object preference, reaction to new environment, reaction to novel objects
**Diseases and pathologic conditions**	-
**Social behaviour**	Allopreening, aggressive behaviours, human-animal interaction, facial and body displays, sexual behaviours, social dynamics, vocalizations

Subjects' living conditions

Table S4. Description of the various living conditions that would apply to the parrots included in the eligible studies that identified potential welfare parameters to assess parrot welfare.

Living condition	Definition
Breeding facility	Parrots living in private or public breeding facilities
Companion animals	Parrots that live with humans or within human social structures where they are provided with some, or all, of their needs. They are considered to play a primarily social role within the household or community (1)
Laboratory animals	Parrot kept for research purposes at universities or laboratories
Multiple	Studies focused on parrots kept in multiple living conditions
Rehabilitation centre	Parrots kept in rehabilitation centres
Shelter	Parrots, previously kept as companion, that lives in shelters
Zoo	Parrots living in zoo with public or private aviaries

Results

Table S5. Total number of studies and number of studies per parrot genus (in parentheses) on welfare-related outcome parameters, grouped according to the parrots' living conditions.

Living condition	Number of Studies	Genera (number of studies)
Breeding facility	6	*Amazona* (n = 1), *Ara* (n = 1), *Nymphicus* (n = 3), *Guaruba* (n = 1)
Companion animals	21	*Agapornis* (n = 1), *Cacatua* (n = 2), Multiple (n = 11), Nymphicus (n = 1), *Psittacus* (n = 7)
Laboratory-kept animals	48	*Amazona* (n = 22), *Melopsittacus* (n = 14), Multiple (n = 1), *Myiopsitta* (n = 2), *Nymphicus* (n=7), *Platycercus* (n=1), *Psittacus* (n=1)
Multiple	1	*Amazona* (n = 1)
Rehabilitation centre	3	*Amazona* (n = 3)
Shelter	2	*Psittacus* (n = 2)
Unknown	7	*Amazona* (n = 1), *Ara* (n = 1), *Myiopsitta* (n = 1), Multiple (n = 2), *Loriculus* (n = 1), *Psittacus* (n = 1)
Zoo	10	*Amazona* (n = 1), *Ara* (n = 4), *Cacatua* (n = 1), *Calyptorhynchus* (n = 1), Multiple (n = 2), *Pyrrhura* (n = 1)

Genera and living conditions represented in the studies

Eligible studies covered 13 genera, of which 10 belonged to the superfamily *Psittaccoidea* and 3 to the superfamily *Cacatuoidea.* None of the studies investigated the welfare of species belonging to the superfamily *Strigopoidea*. *Amazona* was the genus most investigated, representing 29 studies (29.6%), followed by the genera *Melopsittacus*, *Nymphicus*, and *Psittacus* with 14 (14.3%), 11 (11.2%), and 10 studies (10.2%) respectively (Figure S1, Table S8). A lesser number of studies focused on the genera *Ara* (n = 6; 6.1%), *Cacatua* (n = 3; 3.1%), and *Myiopsitta* (n = 3; 3.1%), while six other genera were represented by only one study each (Figure S1, Table S8). Sixteen studies (16.3%) investigated parrot welfare in multiple genera, including those as previously mentioned, as well as the *Eclectus* (6 studies) and *Poicephalus* (6 studies) genera (Figure S1, Table S7 for a full list of species included in the category 'multiple', Table S8).

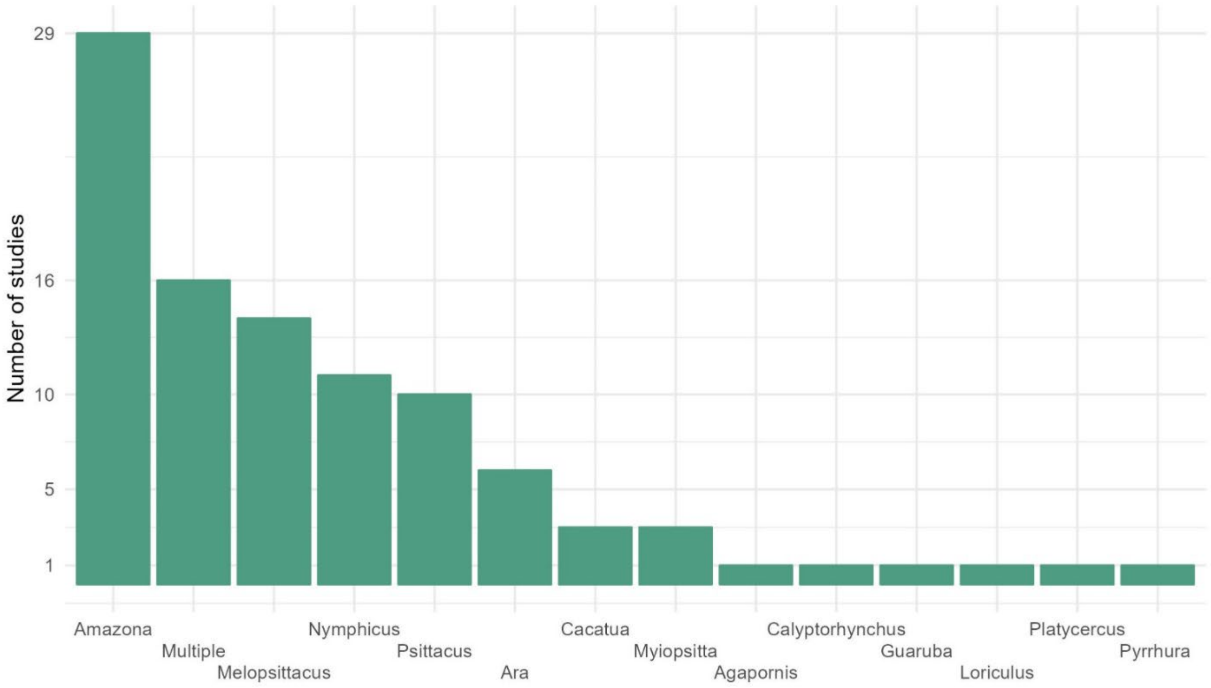

Figure S1. Number of studies identified in the systematic literature search that report on welfare-related outcome measures in parrots, grouped by parrots' genera. The y-axis corresponds to the number of studies reporting on welfare-related outcomes for each parrot genera listed.

Table S6. Number of studies reporting on welfare-related outcome measures for each parrot genus as identified during the systematic literature search. As some studies did not specify the species or genus involved, additional groups were created such as 'cockatoo', 'conure', 'macaw', and 'parakeet' as authors listed the common instead of the scientific name, hindering the ability to attribute the study to a particular genus. Additionally, few studies involving multiple species failed to report on a particular species investigated, classifying these as 'other', 'undetermined' or 'unknown'.

Genus	Number of Studies	Genus	Number of Studies	Genus	Number of Studies	Genus	Number of Studies
Agapornis	6	*Cyanoramphus*	3	*Nadayus*	1	*Psittacara*	1
Alisterus	1	*Deroptyus*	1	*Neophema*	2	*Psittacula*	4
Amazona	10	*Diopsittaca*	2	*Neopsittacus*	1	*Psittaculorostris*	1
Anodorhynchus	2	*Eclectus*	6	*Northiella*	1	*Psittacus*	10
Aprosmictus	1	*Enicognathus*	1	*Nymphicus*	7	*Psitteuteles*	2
Ara	8	*Eolophus*	2	*Opopsitta*	1	*Psittaculorostris*	1
Aratinga	3	*Eos*	2	*Orthopsittaca*	1	*Psittinus*	1
Barnadius	2	*Eupsittula*	1	Other[1]	1	*Purpureicephalus*	1
Bolborhynchus	3	*Forpus*	4	Parakeet[2]	2	*Pyrrhura*	2
Brotogeris	1	*Glossopsitta*	1	*Pionites*	3	*Tanygnathus*	2
Cacatua	6	*Guaruba*	1	*Pionopsitta*	1	*Trichoglossus*	3
Callocephalon	1	*Lathamus*	1	*Pionus*	3	*Triclaria*	1
Chalcopsitta	2	*Lorie*	1	*Platycercus*	3	Undetermined[1]	2
Charmosyna	1	*Lorius*	2	*Poicephalus*	7	Unknown[1]	1
Cockatoo[2]	3	Macaw[2]	2	*Primolius*	1		
Conure[2]	3	*Melopsittacus*	4	*Probosciger*	1		
Coracopsis	1	*Myiopsitta*	4	*Psephotus*	2		
Cyanoliseus	2	*Neopsephotus*	1	*Pseudeos*	2		

[1] *Description reported by the authors.*
[2] *Authors reported common name instead of scientific name.*

Table S7. Absolute and relative (in percentage) number of studies and number of welfare-related outcome measures identified for each parrot genus during the literature search. In addition to the total number of outcome measures, the (absolute and relative) number of significant ($P < 0.05$) and feasible (i.e. not requiring specific skills, expertise or equipment) outcome measures, number of outcome measures specifically studies in companion parrots, and number of significant and feasible outcome measures identified in companion parrots are also presented.

Genera	**Studies (%)**	**Outcomes (%)**	**Significant and feasible outcomes (%)**	***Outcomes (%); only companion***	***Significant and feasible outcomes (%); only companion***
	**Total = 98**	**Total = 1512**	**Total = 572**	**Total = 340**	**Total = 68**
Agapornis	1 (1.2%)	21 (1.39%)	3 (0.52%)	21 (6.18%)	3 (4.41%)
Amazona	29 (29.59%)	320 (21.16%)	128 (22.34%)	12 (3.53%)	/
Ara	6 (6.12%)	142 (9.39%)	72 (12.57%)	/	/
Cacatua	3 (3.06%)	69 (4.56%)	12 (2.09%)	65 (19.12%)	9 (13.24%)
Calyptorhynchus	1 (1.02%)	6 (0.4%)	4 (0.7%)	/	/
Guaruba	1 (1.02%)	3 (0.2%)	1 (0.17%)	/	/
Loriculus	1 (1.02%)	3 (0.2%)	3 (0.52%)	/	/
Melopsittacus	14 (14.28%)	287 (20.63%)	150 (26.22%)	/	/
Multiple	16 (16.32%)	287 (18.98%)	54 (9.42%)	150 (44.12%)	32 (47.06%)
Myiopsitta	3 (3.06%)	48 (3.17%)	9 (1.57%)	/	/
Nymphicus	11 (11.22%)	148 (9.79%)	65 (11.34%)	4 (1.18%)	/
Platycercus	1 (1.02%)	26 (1.72%)	/	/	/
Psittacus	10 (10.20%)	133 (8.80%)	54 (9.44%)	88 (25.88%)	24 (35.29%)
Pyrrhura	1 (1.02%)	19 (1.26%)	17 (2.97%)	/	/

Welfare dimensions represented in the studies

The most common welfare dimensions investigated across all studies, in decreasing order, were ‘body measurements’ (35 studies), followed by ‘social behaviours’ (34 studies), ‘physiological parameters’ (31 studies), ‘maintenance behaviours’ (29 studies), ‘exploratory and foraging behaviours’ (28 studies), ‘locomotor behaviours’ (20 studies), ‘abnormal and fear-related behaviours’ (16 studies), and ‘diseases and pathologic conditions’ (11 studies) (Figure S2 and Table S9).

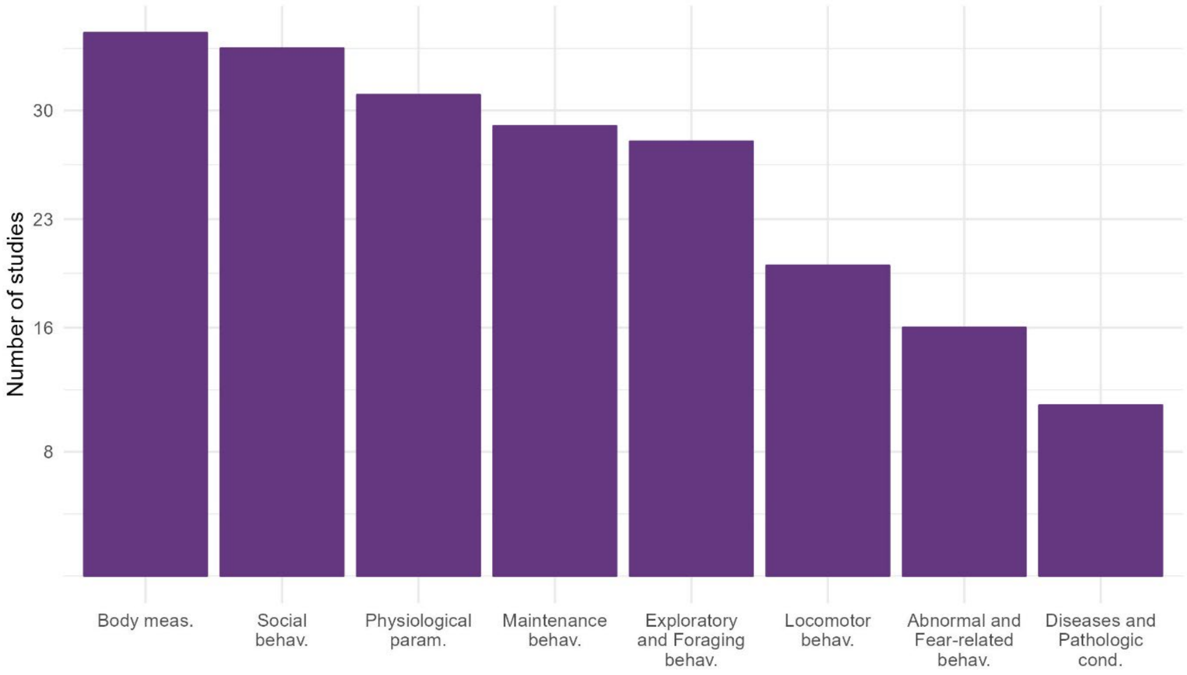

Figure S2. Number of studies reporting on parrot welfare outcome measures as identified during the systematic literature search, grouped by welfare dimension. The y-axis corresponds to the number of studies reporting on at least one welfare-related outcome measures. Behav.=behaviour, meas.=measurements, param.=parameters, cond.=conditions.

Table S8. Absolute and relative (in percentage) number of studies and number of welfare-related outcome measures identified for each welfare dimension identified during the literature search. In addition to the total number of outcome measures, the (absolute and relative) number of significant (*P* < 0.05) and feasible (i.e. not requiring specific skills, expertise or equipment) outcome measures, number of outcome measures specifically studies in companion parrots, and number of significant and feasible outcome measures identified in companion parrots are also presented.

Welfare Dimensions	**Studies (%)**	**Outcomes (%)**	**Significant and feasible outcomes (%)**	***Outcomes (%); only companion***	***Significant and feasible outcomes (%); only companion***
		**Total = 1,512**	**Total = 572**	**Total = 340**	**Total = 68**
Abnormal and fear-related behaviours	16 (7.84%)	157 (10.38%)	87 (15.18%)	34 (10%)	8 (11.76%)
Locomotor behaviours	20 (9.80%)	149 (9.85%)	83 (14.51%)	/	/
Exploratory and foraging behaviours	28 (13.72%)	212 (14.02%)	93 (16.26%)	1 (0.29%)	1 (1.47%)
Diseases and pathologic conditions	11 (5.39%)	61 (4.03%)	/	17 (5%)	/
Maintenance behaviours	29 (14.21%)	185 (12.24%)	87 (15.21%)	6 (1.76%)	2 (2.94%)
Body measurements	35 (17.15%)	316 (20.90%)	80 (13.99%)	231 (67.94%)	51 (75%)
Physiological parameters	31 (15.19%)	192 (12.70%)	/	45 (13.24%)	/
Social behaviours	34 (16.66%)	240 (15.87%)	142 (24.78%)	6 (1.76%)	6 (8.82%)

Welfare categories represented in the studies

The outcome measures were grouped in 35 different welfare categories (Table S4). Of these, 'indirect measures of feather-damaging behaviour' and 'feeding' were covered by the highest number of studies (n = 19), followed by 'self-care' (n = 18) and 'stereotypies' (n = 16) (Table S10). Most other categories were covered by 2 to 9 studies. Of all categories, 'body surface temperature' was the least studied with only one study (Table S10).

Table S10. Significant and feasible outcome measures related to parrot welfare as identified during the systematic literature search, categorised by welfare dimensions and welfare categories. The table includes the number of outcomes associated with each welfare category, the types of outcomes measured, and the parrot genera investigated, along with corresponding studies. The types of outcomes are detailed using abbreviations to denote different measurement methods: D (duration), L (latency), F (frequency), P (percentage), PP (proportion of parrots), and U (unknown type of measurement). In addition to these details, the table includes the feasible risk factors associated with each welfare category, providing insight into potential causes and influences on welfare across various parrot species. For detailed associations between outcomes, risk factors, and their effects, refer to the dataset.

Welfare dimensions	Welfare Categories	Number of Outcomes	Types of outcomes	Feasible risk factors	Genera	Studies
Abnormal and fear-related behaviours	Fear-related	3 (0.2%)	Phobic behaviours (U)	small cage (max. 80 cm 100 cm 120 cm), highest perch lower than eye level, being wild caught	*Psittacus*	(2)
	Incessant screaming	8 (1.40%)	Incessant screaming (D, F)	being pair-housed, small cage	*Melopsittacus*	(3, 4)
	Stereotypies	76 (13.26%)	Oral stereotypies (P), total stereotypies (F, D, P), whole body stereotypies, self-biting, sham/wire chewing (F, D), biting (F), pacing (D, F, PP), route tracing (D, F, PP), spot pecking (D, F)	, < 5 weeks when removed from the nest, brain volume (species), small cage, lack of enrichment, being pair-housed), bird sex, number of birds, unbalanced diet (no fruit or few, no protein supply, inappropriate seed mix, etc), being hand-reared, perches' material (only manufactured), being single-housed	*Amazona*, *Ara*, *Calyptorhynchus*, *Melopsittacus*, "Multiple", *Psittacus*	(2-13)
Exploratory and foraging behaviours	Cognitive	6 (1.05%)	Proactive response towards novel objects, latency to reward during attention bias test, problem-solving skills, responsiveness during discrimination task	Correlation with feather-damaging behaviours, presence of unfamiliar humans-, personality	*Amazona*, *Melopsittacus*, *Psittacus*	(14-16)
	Enrichment interaction	24 (4.19%)	Environment interaction (F, P, PP), object destruction, visit enriched area (F, D), enrichment interaction (F, D, P)	Lack of offered choices, being single-housed, bird sex, lack of enrichments, being hand reared, manual restraint, personality (being explorative)	*Amazona*, *Ara*, "Multiple", *Nymphicus*	(5, 6, 9, 10, 13, 17-21)
	Foraging	16 (2.79%)	Foraging (F, D, P), interaction with food (D), contrafreeloading	Lack of enrichment, personality (individual differences)	*Ara*, *Calyptorhynchus*, *Psittacus*	(11, 13, 22-24)
	Preference	18 (3.14%)	Choice for pellet size or objects colour/size/material, perch diameter/position	Lack of offered choices	*Amazona*, *Loriculus*	(25-28)
	Reaction to new environment	7 (1.22%)	Exploration (D, L), pattern of exploration, total distance/amount squares covered, number of visits of new environments, explorative tendency PCA axis (Results obtained running a Principal Component Analysis)	Correlation with neophobic behaviours, correlation with feather-damaging behaviours, being single-housed	*Melopsittacus*, *Psittacus*	(14, 16, 29)
	Reaction to novel objects	22 (3.84%)	Novel object interaction (D, L), latency to feed in presence of novel object, latency to touch novel object	Being hand-reared, lack of enrichment (physical + foraging), being single-housed), species, being dominant	*Amazona,* "Multiple"	(5, 30-32)
Locomotor behaviours	Flying	26 (4.54%)	Flying (D, F, L, score), escape flight (D)	Small cage, being single-housed, correlation with feather-damaging behaviours, lack of enrichments, bird sex, being hand-reared, insufficient physical activity, wing load (indirect measure of fat mass), personality (being more risk taker)	*Amazona*, *Melopsittacus*, "Multiple", *Pyrrhura*	(5, 9, 29, 33-37)
	Inactivity	9 (1.57%)	Inactivity (PP), stationary position (D)	Lack of enrichment, time of day, correlation with feather-damaging behaviours	*Ara*, *Pyrrhura*	(18, 33)
	Locomotion	34 (5.93%)	Locomotion (F, D), walking (F, D), climbing (D), general activity (D), hopping (D), number of area changes	Being pair-housed, small cage, lack of enrichment, being single-housed), manual restraint	*Amazona*, *Ara*, *Melopsittacus*, *Nymphicus*, *Pyrrhura*,	(4, 5, 8, 10, 13, 18, 19, 21, 33, 36, 38)
	Position occupied in the cage	14 (2.44%)	Standing at the bottom of the cage (F), flights from the perch/wall to the ground (F, D), aggregated flights to the ground (D), fly between walls/perches (F), time spent on the ground, time spent 1m to 2m high, standing at the grid ceiling (F)	Elevated ambient temperature, lack of enrichment, being pair-housed), feeders positioned under the perches in the cage	*Ara*, *Melopsittacus*, *Nymphicus, Pyrrhura*,	(10, 21, 33, 34, 39)
Maintenance behaviours	Drinking	2 (0.35%)	Water intake	Diet based only on seeds, being exposed to artificial light at night	*Amazona, Melopsittacus*	(40, 41)
	Feeding	31 (5.41%)	Food consumption (D), food intake, time spent feeding per day (P), feeding (D, F), visit to feeding dish (F), ratio consumption (grams per bird)	Being single-housed, elevated temperatures, food preference, lack of enrichment (physical/foraging), personality (being more vigilant), time of day, manual restraint, small cage, bird sex, courtship feeding, flight activity (frequency), diet based only on seeds, being exposed to artificial light at night	*Amazona*, *Ara*, *Melopsittacus*, *Myiopsitta*, Nymphicus, *Psittacus*	(18-20, 23, 29, 34, 36, 39-46)
	Self-care	42 (7.33%)	Preening (D, L, P), bathing (D, L), tail wagging (F), puffing up the feathers (F), scratching (PP), cleaning the beak (PP), wing stretch (F)	Reduced flight ability, being hand reared or sold before the end of weaning, lack of bathing opportunities, correlation with feather damaging behaviours, lack of enrichment, small cage, bird sex, time of day, being pair-housed), being single-housed	*Amazona*, *Ara*, *Melopsittacus*, "Multiple", *Psittacus*, *Pyrrhura*	(2-5, 8-10, 18, 20, 29, 33, 36, 42)
	Resting	12 (2.09%)	Resting/sleeping (D, F, P)	Lack of enrichment, small cage, being pair-housed, manual restraint, time of day, being single-housed	*Amazona*, *Ara*, *Melopsittacus*, *Nymphicus*	(4, 5, 13, 19, 20, 42, 44)
Body measurements	Body condition	12 (2.09%)	Body mass, body weight, chest girth	exposure to artificial light at night, lack of exercise, diet based only on seeds, correlation with flight activity, correlation with feeding duration, correlation with wing load (indirect measure of fat mass), bird sex, general activity	*Amazona, Ara, Melopsittacus*	(34, 41, 46-48)
	Indirect measures of feather-damaging behaviour	66 (12.04%)	Presence/absence of feather damages, plumage score	Diet needing extensive handling, ≥ 1 vacation per year taken by owners , age, small cage, absence of foraging/chewable devices, receiving command training, personality (being proactive), distance of the cage from the door, variety of the diet, heritability, hours spent outside of the cage, hours of sleep, lack of enrichments, length of ownership, lives with others parrots, position of the cage, bird sex, no toys/only one toy in the cage, inability to fly, other non-bird companion animals (protective), out of the cage for more than 8 h, owner type (Shelter, Woman, Man, Family), being acquired from a pet shop, being hand reared, being sold before the end of weaning, being rescued or rehomed, separation anxiety, species, sprayed with water daily, time of human/bird interaction per day	*Agapornis, Amazona*, *Cacatua*, *Guaruba*, "Multiple", *Psittacus*	(2, 7, 8, 12, 14, 22, 49-60)
Social behaviours	Aggressive	18 (3.14%)	Total aggressive behaviours (F, D), female/male aggressive behaviours (F), aggressive behaviours toward non-mates (F), wins/losses after agonistic interactions (F), number of chicks killed	Dominance rank, agonistic interactions, partner/non-partner affiliation, manual restraint, bird sex, mate/non-mate, being pair-housed	*Amazona*, *Melopsittacus*, *Myiopsitta*, *Nymphicus*	(19, 61-66)
	Allopreen	12 (2.09%)	Male/female allopreen (F), male/female allopreen solicitation (F), total allopreening (D, PP, P)	Sex of the receiver, small cage, correlation with feather damaging behaviours, lack of enrichments, type of physical enrichment, preference for partner	*Amazona*, *Ara*, *Melopsittacus*, *Nymphicus*, *Pyrrhura*	(18, 20, 33, 36, 61, 62)
	Sexual behaviours	19 (3.31%)	Female/male courting (D, F), intra-pair interaction (D), number of approaches toward mates, synchronicity, female/male copulation (F), female solicits copulation (F), sexually active (U), sexual behaviours (P)	Correlation with feather-damaging behaviours, small cage, being pair-housed, bird sex, comparison between mate and non-mate, lack of physical, foraging, and cognitive enrichments, < 2 weeks in the nest from hatching or hatched from egg incubator	*Ara, Melopsittacus*, *Nymphicus*, *Psittacus*, *Pyrrhura*	(2, 4, 13, 33, 62)
	Social dynamics	21 (3.66%)	Reaction towards other individuals (F), coo-feeding (F), food steeled (F, number of parrots), social interactions (PP), time spent next to each other, approach unfamiliar bird (L), dominance rank, physical distance between subjects (score)	Manual restraint, kinship, age, aggressiveness, time of day, bird sex, lack of enrichment, being single-housed, small cage, lack of mate	*Ara*, *Melopsittacus*, *Nymphicus*	(18, 19, 29, 61, 62, 65, 67)
	Vocalisations	23 (4.01%)	Calm vocalisation (F), singing (D, F), vocalisation (D, F, P), playback response (F)	Affiliation with other birds, correlation with feather damaging behaviours, correlation with accepting food from humans, lack of enrichments, being pair-housed, having reduced flight ability, being separated from the flock, being single housed	*Amazona*, *Ara*, *Melopsittacus*, "Multiple", *Myiopsitta, Nymphicus*, *Pyrrhura*	(4, 9, 10, 20, 29, 33, 38, 64)
	Human-animal interaction	34 (6.28%)	Attention bias, , yawning after handling (L, F), response to unfamiliar/familiar handler (score), human aversion score, seeking behaviour toward humans (F), human-direct aggressiveness (yes/no), approach to humans (yes/no), food acceptance (yes/no, score), response to human contacts (score), resistance to being picked up (yes/no), latency to approach humans, begging once adult (yes/no), selective toward humans, tendency to anthropomorphise PCA axis*, vocalise during restrain (D, L, F), learned vocalisation (F), long distance contact call (F)	Unfamiliar human presence, lack of human-animal interaction, inappropriate interaction, neophilia score, lack of neonatal human handling, lack of enrichment, being hand-reared, mouth to beak feeding, human food consumption, owner gender, correlation with feather-damaging behaviours, being single-housed	*Amazona*, *Ara*, *Melopsittacus*, "Multiple", *Psittacus*	(2, 5, 9, 14, 15, 31, 37, 68-73)
	Facial and body displays	13 (2.27%)	Crown, nape, lower/upper mandible, cheek feathers ruffling (scan), nape/crown feather height, erected crest (D), blushing around eyes, beak grinding (F)	Correlation with positive human-parrot interaction, arousal level, being separated from the flock	*Ara, Cacatua*, *Melopsittacus, Nymphicus*	(36, 67, 71, 73, 74)

Table S11. Significant and not feasible outcome measures related to parrot welfare as identified during the systematic literature search, categorised by welfare dimensions and welfare categories. The table reports non-feasible outcome measures grouped by welfare dimensions and categories, showing the types of not feasible outcomes collected, associated feasible risk factors, and the genera of parrots studied, along with their corresponding studies. For detailed associations between outcomes, risk factors, and their effects, refer to the dataset.

Welfare dimension	Welfare categories	Types of not feasible outcomes	Feasible risk factors	Genera	Studies
**Diseases and pathologic conditions**	Health	Presence of atherosclerosis, severity of atherosclerosis lesions, hepatic haemosiderosis, lipid accumulation lesions, prevalence of lipoid pneumonia, presence of ingluvioliths, health conditions, prevalence of hepatic lipidosis, presence of viral diseases	Increase age, being hand-reared using a tube, diet based only on seeds, species, fibres ingestion (bird sex, age), chicks artificial feeding method	*Amazona, "Multiple", Myiopsitta, Nymphicus, Psittacus*	(2, 46, 75-81)
**Body measurements**	Feathers Colour	Cheek, front and crown feathers chroma, front feathers hue, crown feathers luminance, cheek feathers structural colours	Manual restraint, correlation with carotenoid levels	*Platycercus*	(82)
**Physiological parameters**	Lipids-related	Cholesterol concentration, triglyceride concentration, high-density lipoprotein-cholesterol (HDL-C) concentration, low-density lipoprotein-cholesterol (LDL-C) concentration, ratio of total cholesterol and HDL-C,	diet based only on seeds, diet with high cholesterol concentration, diet based on pellet (preventive) , bird sex, lack of exercise	*Amazona, Ara*	(46-48)
	Immune System-related	Humoral response to vaccination, delayed-type hypersensitivity (DTH) response, ratio of heterophils and lymphocytes, leukocytes, lymphocytes, and monocytes count	Lack of human neonatal handling, diet based only seeds, lack of carotenoid supplementation	*Amazona*, *Platycercus*	(46, 69, 82)
	Metabolic	Digestibility of crude fibres, crude proteins and dry matters, daily energy expenditure, malondialdehyde (MDA) concentration	Age, diet based only on seeds, diet based on pellets (preventive) lack of exercise, courtship feeding, correlation with weight and wing load (indirect measure of fat mass)	*Amazona*, Melopsittacus	(34, 35, 83, 84)
	Stress-related	Corticosterone excreta metabolites concentration, plasma corticosterone, cortisol excreta metabolites concentration,	Age, agonistic interactions, exposure to artificial light at night, increase in the dominance rank, bird sex, correlation with feather-damaging behaviour, being hand-reared, correlation with foraging time, manual restraint, lack of human neonatal handling, living conditions (wild > zoo > breeding centre > companions), being single-housed, correlation with locomotor behaviours before implementing enrichment, correlation with object interaction before implementing enrichment, being wild caught	*Agapornis, Amazona*, *Ara*, *Melopsittacus*, "Multiple", *Nymphicus*, *Platycercus, Psittacus*	(10, 19, 41, 60, 66, 69, 82, 85-88)
	Vitamin D-related	Calcifediol concentration, plasma vitamin-D concentration, plasma Ca+ concentration, plasma Mg+ concentration,	Indoor housing, lack of exposure to UV light	*Amazona*	(89, 90)
	Others	DNA damage, telomere length, basal glucose concentration, respiration rate, aortic pressure gradient and speed, haemoglobulin concentration, aspartate amino transferase concentration, glucose concentration,	Increase age, unbalanced diet, type of diet, lack of human neonatal handling, being single-housed, correlation with weight, correlation with activity level	*Amazona*, *Ara*, *Melopsittacus*, *Psittacus*	(46, 47, 68, 69, 84, 91, 92)

Table S12. Significant and feasible welfare-related outcomes measures reported in companion parrots specifically. The table lists welfare categories, types of outcomes observed, the number and percentage of outcomes, risk factors contributing to the increase or decrease of the observed outcome measure (↑/↓), the genera of parrots studied, and the types of interventions used.

Welfare category	Types of outcomes	Number of outcomes (%)	Risk factors	Genera (number of outcomes)	Intervention (number of outcomes)
**Indirect measures of feather damaging behaviours**	Presence/absence of feather damages, plumage score	51 (75%)	Due to high number, see Table S13	*Agapornis* (3), *Cacatua* (9), *Psittacus* (10), Multiple (29)	Video-Analysis (5), Clomipramine treatment (2), Questionnaire (39), Check-up at veterinary clinic (3)
**Fear-related**	Phobic behaviour ↑	1 (1.47%)	Being wild caught	*Psittacus*	Questionnaire
		2 (2.94%)	Perches lower than eye level		
			Small cage (max. 80 cm x 100 cm x 120 cm)		
**Foraging**	Contra-freeloading as foraging enrichment ↑↓	1 (1.47%)	Individuality	*Psittacus*	Contra-freeloading test
**Human-Animal Interaction**	Anthropomorphising ↑	1 (1.47%)	Female ownership	Multiple	Questionnaire
	Begging once adult ↑	3 (4.6%)	Being hand-reared	*Psittacus*	
			Human mouth to beak feeding		
			Human leftover consumption		
	Selective towards humans ↑	1 (1.47%)	Being hand-reared		
**Stereotypies**	Multiple Stereotypies ↑	1 (1.47%)	Inappropriate diet	*Psittacus*	Questionnaire
		1 (1.47%)	Only manufactured perches in the cage		
		1 (1.47%)	Being removed from the nest before 5 weeks of age		
	Whole Body Stereotypies↑	1 (1.47%)	Positive correlation with brain volume (species)	Multiple	Questionnaire
	Oral Stereotypies ↑	1 (1.47%)			
**Sexual behaviours**	Sexual activity ↓	1 (1.54%)	Being removed from the nest before 2 weeks of age or hatched from egg incubator	*Psittacus*	Questionnaire
**Self-care**	Preening ↑	1 (1.54%)	Being hand-reared	*Psittacus*	Questionnaire
		1 (1.54%)	Being sold before the end of weaning		

Outcomes related to feather-damaging behaviours

From the 690 significant results, 70 outcomes, collected from 19 different studies, were related to feather-damaging behaviours. Most of the outcomes were collected from companion animals (n = 51), followed by parrots kept in laboratories (n = 11), shelter parrots (n = 6) and parrots kept in breeding and rehab centres (both n = 1). Most of the outcomes (n = 22) were associated with risk factors belonging to demographic characteristics such as age, sex, and species

(Table S13). The lack of enrichment opportunities (14 outcomes) and factors related to the human-animal relationship (15 outcomes) turned out to also be common potential risk factors for feather-damaging behaviour (Table S13).

Table S13. Summary of risk factors associated with the emergence of feather-damaging behaviour in parrots as identified during the systematic literature search. The risk factors are organised into categories. For each risk factor, the table shows the number of outcomes related to feather damaging behaviour, the number of studies, the parrot genera studied, and the living conditions of the parrots under investigation.

Category	Risk factor	Outcomes	Number of Studies	Genera	Living condition (number of studies)
**Ease of Movement**	Inability to fly	1	1	*Psittacus*	Companion
**Demographic**	Increasing age	6	4	*Agapornis*, Multiple, *Psittacus*	Companion
	Heritability	2	1	*Amazona*	Lab
	Sex	3	3	*Amazona*, *Cacatua*, Multiple	Companion (2), Lab (1)
	Being rescued	1	1	Multiple	Companion
	Species	9	3	Multiple	Companion
	Diet needing extensive handling (species)	1	1	Multiple	Companion
**Enrichment**	Lack of chewable devices	1	1	Multiple	Companion
	Lack of foraging devices	1	1	Multiple	Companion
	Hour spent outside of the cage	1	1	Multiple	Companion
	Lack of foraging + physical enrichment	2	1	*Amazona*	Lab
	Lack of foraging + human and physical enrichment	4	1	*Amazona*	Lab
	Lack foraging enrichment	3	1	*Psittacus*	Shelter
	No toys/only one toy in the cage	1	1	*Psittacus*	Companion
	Out of the cage > 8 h	1	1	Multiple	Companion
**Good Human and Animal relationship**	≥ 1 vacation per year taken by owners	2	1	*Cacatua, Psittacus*	Companion
	Receiving command training	1	1	*Cacatua*	Companion
	Negatively correlated with length of ownership	1	1	*Psittacus*	Companion
	Owner type (shelter > woman > man > family)	1	1	Multiple	Companion
	Being bought from a pet shop	1	1	*Cacatua*	Companion
	Rearing method (being hand-reared, being sold before the end of weaning, lack of human neonatal handling)	6	4	*Amazona*, *Agapornis* Multiple, *Psittacus*	Companion (4), Lab (1)
	Separation anxiety	2	2	*Agapornis*, Multiple	Companion
	Negatively correlated with time of human-parrot interaction per day	1	1	Multiple	Companion
**Good Housing**	Cage volume > 2 m³	1	1	*Cacatua*	Companion
	Increase distance of the cage from the door	1	1	*Amazona*	Lab
	Location of the cage against >= 1 m wall	1	1	*Cacatua*	Companion
**Enrichment+Personality**	Lack of enrichments + neuroticism score	1	1	*Psittacus*	Lab
**Maintenance**	≥ 8 h of sleep	1	1	*Psittacus*	Companion
	Being sprayed with water daily	1	1	*Cacatua*	Companion
**Good Feeding**	Fed with only seed or pellet	1	1	Multiple	Companion
**Personality**	Coping style (being proactive)	3	1	*Psittacus*	Shelter
**Physiological**	Increased adrenocortical activity	1	1	*Guaruba*	Breeding Centre
**Social**	Living with other parrots	1	1	Multiple	Companion
	Living without other non-bird companion animals	1	1	Multiple	Companion

References

1. Farnworth MJ. Pets. In: Vonk J, Shackelford T, editors. Encyclopedia of Animal Cognition and Behavior. Cham: Springer International Publishing; 2018. p. 1-13. https://10.1007/978-3-319-47829-6_367-1

2. Schmid R, Doherr MG, Steiger A. The influence of the breeding method on the behaviour of adult African grey parrots (Psittacus erithacus). Applied Animal Behaviour Science. 2006;98(3/4):293-307.https://doi.org/10.1016/j.applanim.2005.09.002

3. Polverino G, Manciocco A, Alleva E. Effects of spatial and social restrictions on the presence of stereotypies in the budgerigar (Melopsittacus undulatus): a pilot study. Ethology Ecology & Evolution. 2012;24(1):39-53.https://doi.org/10.1080/03949370.2011.582045

4. Polverino G, Manciocco A, Vitale A, Alleva E. Stereotypic behaviours in Melopsittacus undulatus: behavioural consequences of social and spatial limitations. Applied Animal Behaviour Science. 2015;165:143-55.https://doi.org/10.1016/j.applanim.2015.02.009

5. Meehan CL, Garner JP, Mench JA. Isosexual pair housing improves the welfare of young Amazon parrots. Applied Animal Behaviour Science. 2003;81(1):73-88.https://doi.org/10.1016/S0168-1591(02)00238-1

6. Meehan CL, Garner JP, Mench JA. Environmental enrichment and development of cage stereotypy in Orange-winged Amazon parrots (Amazona amazonica). Developmental psychobiology. 2004;44(4):209-18.https://doi.org/10.1002/dev.20007

7. Garner JP, Meehan CL, Famula TR, Mench JA. Genetic, environmental, and neighbor effects on the severity of stereotypies and feather picking in Orange-winged Amazon parrots (Amazona amazonica): an epidemiological study. Applied Animal Behaviour Science. 2006;96(1/2):153-68.https://doi.org/10.1016/j.applanim.2005.09.009

8. Cussen VA, Mench JA. The Relationship between Personality Dimensions and Resiliency to Environmental Stress in Orange-Winged Amazon Parrots (Amazona amazonica), as Indicated by the Development of Abnormal Behaviors. PloS one. 2015;10(6):e0126170.https://doi.org/10.1371/journal.pone.0126170

9. Williams I, Hoppitt W, Grant R. The effect of auditory enrichment, rearing method and social environment on the behavior of zoo-housed psittacines (Aves: Psittaciformes); implications for welfare. Applied Animal Behaviour Science. 2017;186:85-92.https://doi.org/10.1016/j.applanim.2016.10.013

10. Almeida ACd, Palme R, Moreira N. How environmental enrichment affects behavioral and glucocorticoid responses in captive blue-and-yellow macaws (Ara ararauna). Applied Animal Behaviour Science. 2018;201:125-35.https://doi.org/10.1016/j.applanim.2017.12.019

11. Fangmeier ML, Burns AL, Melfi VA, Meade J. Foraging enrichment alleviates oral repetitive behaviors in captive red-tailed black cockatoos (Calyptorhynchus banksii). Zoo biology. 2020;39(1):3-12.https://doi.org/10.1002/zoo.21520

12. Mellor EL, Kinkaid HKM, Mendl MT, Cuthill IC, van Zeeland YRA, Mason GJ. Nature calls: intelligence and natural foraging style predict poor welfare in captive parrots. Proceedings of the Royal Society B-Biological Sciences. 2021;288(1960).https://doi.org/10.1098/rspb.2021.1952

13. Miglioli A, Vasconcellos AdS. Can behavioural management improve behaviour and reproduction in captive blue-and-yellow macaws (Ara ararauna)? Applied Animal Behaviour Science. 2021;241.https://doi.org/10.1016/j.applanim.2021.105386

14. van Zeeland YRA, Aa MMJAvd, Vinke CM, Lumeij JT, Schoemaker NJ. Behavioural testing to determine differences between coping styles in Grey parrots (Psittacus erithacus erithacus) with and without feather damaging behaviour. Applied Animal Behaviour Science. 2013;148(3):218-31.https://doi.org/10.1016/j.applanim.2013.08.004

15. Cussen VA, Mench JA. Personality predicts cognitive bias in captive psittacines, Amazona amazonica. Animal Behaviour. 2014;89:123-30.https://doi.org/10.1016/j.anbehav.2013.12.022
16. Medina-Garcia A, Jawor JM, Wright TF. Cognition, personality, and stress in budgerigars, Melopsittacus undulatus. Behavioral Ecology. 2017;28(6):1504-16.https://doi.org/10.1093/beheco/arx116
17. Webb NV, Famula TR, Millam JR. The effect of rope color, size and fray on environmental enrichment device interaction in male and female Orange-winged Amazon parrots (Amazona amazonica). Applied Animal Behaviour Science. 2010;124(3/4):149-56.https://doi.org/10.1016/j.applanim.2010.02.013
18. Reimer J, Maia CM, Santos EF. Environmental Enrichments for a Group of Captive Macaws: Low Interaction Does Not Mean Low Behavioral Changes. Journal of applied animal welfare science : JAAWS. 2016;19(4):385-95.https://doi.org/10.1080/10888705.2016.1175944
19. Turpen KK, Welle KR, Trail JL, Patel SD, Allender MC. Establishing stress behaviors in response to manual restraint in cockatiels (Nymphicus hollandicus). Journal of Avian Medicine and Surgery. 2019;33(1):38-45.https://doi.org/10.1647/2017-315
20. Ramos GAP, Azevedo CS, Jardim THA, Sant'Anna AC. Temperament in Captivity, Environmental Enrichment, Flight Ability, and Response to Humans in an Endangered Parrot Species. Journal of applied animal welfare science : JAAWS. 2021;24(4):379-91.https://doi.org/10.1080/10888705.2020.1765367
21. Stevens A, Doneley R, Cogny A, Phillips CJC. The effects of environmental enrichment on the behaviour of cockatiels (Nymphicus hollandicus) in aviaries. Applied Animal Behaviour Science. 2021;235.https://doi.org/10.1016/j.applanim.2020.105154
22. Lumeij JT, Hommers CJ. Foraging 'enrichment' as treatment for pterotillomania. Applied Animal Behaviour Science. 2008;111(1/2):85-94.https://doi.org/10.1016/j.applanim.2007.05.015
23. van Zeeland YRA, Schoemaker NJ, Ravesteijn MM, Mol M, Lumeij JT. Efficacy of foraging enrichments to increase foraging time in Grey parrots (Psittacus erithacus erithacus). Applied Animal Behaviour Science. 2013;149(1/4):87-102.https://doi.org/10.1016/j.applanim.2013.09.005
24. Smith GE, Greene D, Hartsfield LA, Pepperberg IM. Initial evidence for eliciting contrafreeloading in grey parrots (Psittacus erithacus) via the opportunity for playful foraging. Journal of comparative psychology (Washington, DC : 1983). 2021;135(4):516-33.https://doi.org/10.1037/com0000295
25. Kim LC, Garner JP, Millam JR. Preferences of Orange-winged Amazon parrots (Amazona amazonica) for cage enrichment devices. Applied Animal Behaviour Science. 2009;120:216-23.https://doi.org/10.1016/j.applanim.2009.06.006
26. Rozek JC, Danner LM, Stucky PA, Millam JR. Over-sized pellets naturalize foraging time of captive Orange-winged Amazon parrots (Amazona amazonica). Applied Animal Behaviour Science. 2010;125(1/2):80-7.https://doi.org/10.1016/j.applanim.2010.03.001
27. Rozek JC, Millam JR. Preference and motivation for different diet forms and their effect on motivation for a foraging enrichment in captive Orange-winged Amazon parrots (Amazona amazonica). Applied Animal Behaviour Science. 2011;129(2/4):153-61.https://doi.org/10.1016/j.applanim.2010.11.009
28. Andrýsek J, Chládek G, Javorová J, Velecká M, Večeřa M, Falta D. Resting behaviour of parrots Loriculus galgulus. Acta Universitatis Agriculturae et Silviculturae Mendelianae Brunensis. 2014;62(1):17-22.https://doi.org/10.11118/actaun201462010017
29. Nicol CJ, Pope SJ. A comparison of the behaviour of solitary and group-housed budgerigars. Animal Welfare. 1993;2(3):269-77.https://doi.org/10.1017/S0962728600015918
30. Mettke-Hofmann C, Winkler H, Leisler B. The Significance of Ecological Factors for Exploration and Neophobia in Parrots. Ethology. 2002;108(3):249-72.https://doi.org/10.1046/j.1439-0310.2002.00773.x
31. Meehan CL, Mench JA. Environmental enrichment affects the fear and exploratory responses to novelty of young Amazon parrots. Applied Animal Behaviour Science. 2002;79(1):75-88.https://doi.org/10.1016/S0168-1591(02)00118-1

32. Fox RA, Millam JR. Novelty and individual differences influence neophobia in orange-winged Amazon parrots (Amazona amazonica). Applied Animal Behaviour Science. 2007;104(1/2):107-15.https://doi.org/10.1016/j.applanim.2006.04.033
33. VanHoek CS, King CE. Causation and influence of environmental enrichment on feather picking of the crimson-bellied conure (Pyrrhura perlata perlata). Zoo biology. 1997;16(2):161-72.https://doi.org/10.1002/(SICI)1098-2361(1997)16:2<161::AID-ZOO6>3.0.CO;2-8
34. Schnegg A, Gebhardt-Henrich SG, Keller P, Visser GH, Steiger A. Feeding behaviour and daily energy expenditure of domesticated budgerigars (Melopsittacus undulatus): influence of type of housing and vertical position of the feeder. Applied Animal Behaviour Science. 2007;108(3/4):302-12.https://doi.org/10.1016/j.applanim.2007.01.008
35. Larcombe SD, Coffey JS, Bann D, Alexander L, Arnold KE. Impacts of dietary antioxidants and flight training on post-exercise oxidative damage in adult parrots. Comparative biochemistry and physiology Part B, Biochemistry & molecular biology. 2010;155(1):49-53.https://doi.org/10.1016/j.cbpb.2009.09.009
36. Phillips CJC, Farrugia C, Lin C, Mancera K, Doneley B. The effect providing space in excess of standards on the behaviour of budgerigars in aviaries. Applied Animal Behaviour Science. 2018;199:89-93.https://doi.org/10.1016/j.applanim.2017.10.015
37. Franzone V, Ramos GdAP, de Lima Kascher LK, de Azevedo CS, Sant'Anna AC. Flight capacity and human aversion in captive Amazon parrots: Related factors and the effects of pre-releasing training. Applied Animal Behaviour Science. 2022;256:105772.https://doi.org/10.1016/j.applanim.2022.105772
38. Lievin-Bazin A, Pineaux M, Clerc O, Gahr M, von Bayern AMP, Bovet D. Emotional responses to conspecific distress calls are modulated by affiliation in cockatiels (Nymphicus hollandicus). PloS one. 2018;13(10).https://doi.org/10.1371/journal.pone.0205314
39. Carvalho TSG, Zangeronimo MG, Saad CEP, Alvarenga RR, Assis VDL, Pereira VM, et al. Behaviour of cockatiels (Nymphicus hollandicus) at two temperatures in captivity. Arquivo Brasileiro de Medicina Veterinária e Zootecnia. 2015;67(6):1669-74.https://doi.org/10.1590/1678-4162-7892
40. Kalmar ID, Veys AC, Geeroms B, Reinschmidt M, Waugh D, Werquin G, et al. Effects of segregation and impact of specific feeding behaviour and additional fruit on voluntary nutrient and energy intake in yellow-shouldered amazons (Amazona barbadensis) when fed a multi-component seed diet ad libitum. Journal of animal physiology and animal nutrition. 2010;94(6):e383-92.https://doi.org/10.1111/j.1439-0396.2010.01026.x
41. Malek I, Haim A, Izhaki I. Melatonin mends adverse temporal effects of bright light at night partially independent of its effect on stress responses in captive birds. Chronobiology international. 2020;37(2):189-208.https://doi.org/10.1080/07420528.2019.1698590
42. Murphy SM, Braun JV, Millam JR. Bathing behavior of captive Orange-winged Amazon parrots (Amazona amazonica). Applied Animal Behaviour Science. 2011;132(3/4):200-10.https://doi.org/10.1016/j.applanim.2011.04.010
43. Beaufrere H, Nevarez JG, Wakamatsu N, Clubb S, Cray C, Tully TN. Experimental Diet-Induced Atherosclerosis in Quaker Parrots (Myiopsitta monachus). Veterinary Pathology. 2013;50(6):1116-26.https://doi.org/10.1177/0300985813488958
44. Carvalho TSG, Saad CEP, Alvarenga RR, Ferreira WM, Assis VDL, Pereira VM, et al. Use of collard green stalks as environmental enrichment for cockatiels (Nymphicus hollandicus) kept in captivity. Arquivo Brasileiro De Medicina Veterinaria E Zootecnia. 2017;69(3):718-24.https://doi.org/10.1590/1678-4162-8988
45. Naves DAS, Carvalho TSG, Zangeronimo MG, Saad CEP, Scalon JD. Food preferences of cockatiel chicks (Nymphicus hollandicus) in captivity. Arquivo Brasileiro de Medicina Veterinária e Zootecnia. 2017;69(3):683-6.https://doi.org/10.1590/1678-4162-9430
46. Di Santo LG, Braos LB, Kawanami AE, Oliveira JP, Cruz NRN, Mendonça FS, et al. Feed processing effects on digestibility, palatability, excreta fermentation products and blood parameters in blue-fronted amazon parrots (Amazona aestiva)(†). Journal of animal physiology and animal nutrition. 2019;103(1):339-53.https://doi.org/10.1111/jpn.13011

47. Veloso RR, Jr., Sakomura NK, Kawauchi IM, Malheiros EB, Carciofi AC. Effects of food processing and fibre content on the digestibility, energy intake and biochemical parameters of Blue-and-gold macaws (Ara ararauna L. - Aves, Psittacidae). Journal of animal physiology and animal nutrition. 2014;98(2):251-61.https://doi.org/10.1111/jpn.12075
48. Gustavsen KA, Stanhope KL, Lin AS, Graham JL, Havel PJ, Paul-Murphy JR. EFFECTS OF EXERCISE ON THE PLASMA LIPID PROFILE IN HISPANIOLAN AMAZON PARROTS (AMAZONA VENTRALIS) WITH NATURALLY OCCURRING HYPERCHOLESTEROLEMIA. Journal of zoo and wildlife medicine : official publication of the American Association of Zoo Veterinarians. 2016;47(3):760-9.https://doi.org/10.1638/2015-0192.1
49. Meehan CL, Millam JR, Mench JA. Foraging opportunity and increased physical complexity both prevent and reduce psychogenic feather picking by young Amazon parrots. Applied Animal Behaviour Science. 2003;80(1):71-85.https://doi.org/10.1016/S0168-1591(02)00192-2
50. Fox RA, Millam JR. The effect of early environment on neophobia in orange-winged Amazon parrots (Amazona amazonica). Applied Animal Behaviour Science. 2004;89(1/2):117-29.https://doi.org/10.1016/j.applanim.2004.05.002
51. Seibert LM, Crowell-Davis SL, Wilson GH, Ritchie BW. Placebo-controlled clomipramine trial for the treatment of feather picking disorder in cockatoos. Journal of the American Animal Hospital Association. 2004;40(4):261-9.https://doi.org/10.5326/0400261
52. Kinkaid HYM, Mills DS, Nichols SG, Meagher RK, Mason GJ. Feather-damaging Behaviour in Companion Parrots: An Initial Analysis of Potential Demographic Risk Factors. Avian Biology Research. 2013;6(4):289-96.https://doi.org/10.3184/175815513X13803574144572
53. van Zeeland YRA, Bergers MJ, van der Valk L, Schoemaker NJ, Lumeij JT. Evaluation of a novel feather scoring system for monitoring feather damaging behaviour in parrots. Veterinary Journal. 2013;196(2):247-52.https://doi.org/10.1016/j.tvjl.2012.08.020
54. Gaskins LA, Hungerford L. Nonmedical Factors Associated With Feather Picking in Pet Psittacine Birds. Journal of Avian Medicine and Surgery. 2014;28(2):109-17.https://doi.org/10.1647/2012-073R
55. Jayson SL, Williams DL, Wood JLN. Prevalence and risk factors of feather plucking in African grey parrots (Psittacus erithacus erithacus and Psittacus erithacus timneh) and cockatoos (Cacatua spp.). Journal of Exotic Pet Medicine. 2014;23(3):250-7.https://doi.org/10.1053/j.jepm.2014.06.012
56. Costa P, Macchi E, Tomassone L, Ricceri F, Bollo E, Scaglione FE, et al. Feather picking in pet parrots: sensitive species, risk factor and ethological evidence. Italian Journal of Animal Science. 2016;15(3):473-80.https://doi.org/10.1080/1828051X.2016.1195711
57. Acharya R, Rault JL. Risk factors for feather-damaging behavior in companion parrots: a social media study. Journal of Veterinary Behavior: Clinical Applications and Research. 2020;40:43-9.https://doi.org/10.1016/j.jveb.2020.07.003
58. Sinhorini JA, Pizzutto CS, Palme R. ACTH Stimulation Induced Self-Mutilation Behavior in the Golden Conure (Guaruba guarouba). Animals : an open access journal from MDPI. 2020;10(3).https://doi.org/10.3390/ani10030418
59. Ebisawa K, Nakayama S, Pai CY, Kinoshita R, Koie H. Prevalence and risk factors for feather-damaging behavior in psittacine birds: Analysis of a Japanese nationwide survey. PloS one. 2021;16(7).https://doi.org/10.1371/journal.pone.0254610
60. Ebisawa K, Kusuda S, Nakayama S, Pai C, Kinoshita R, Koie H. Effects of rearing methods on feather-damaging behavior and corticosterone metabolite excretion in the peach-faced lovebird (Agapornis roseicollis Vieillot). Journal of Veterinary Behavior. 2022;54:28-35.https://doi.org/10.1016/j.jveb.2022.07.002
61. Seibert LM, Crowell-Davis SL. Gender effects on aggression, dominance rank, and affiliative behaviors in a flock of captive adult cockatiels (Nymphicus hollandicus). Applied Animal Behaviour Science. 2001;71(2):155-70.https://doi.org/10.1016/S0168-1591(00)00172-6
62. Spoon TR, Millam JR, Owings DH. Variation in the stability of cockatiel (Nymphicus hollandicus) pair relationships: The roles of males, females, and mate compatibility. Behaviour. 2004;141:1211-34.https://doi.org/10.1163/1568539042729711

63. Banaszewska D, Ostrowski D, Biesiada-Drzazga B, Andraszek K, Górski K, Flis-Chruściel M. The influence of different breeding units and breeding systems on reproductive results of cockatiels (Nymphicus hollandicus). Acta Scientiarum Polonorum - Zootechnica. 2015;14(1):15-24
64. Hobson EA, John DJ, McIntosh TL, Avery ML, Wright TF. The effect of social context and social scale on the perception of relationships in monk parakeets. Current Zoology. 2015;61(1):55-69.https://doi.org/10.1093/czoolo/61.1.55
65. Duggan MR, Lee-Soety JY, Anderson MJ. Personality types in Budgerigars, Melopsittacus undulatus. Behav Processes. 2017;138:34-40.https://doi.org/10.1016/j.beproc.2017.02.003
66. Matos LSD, Palme R, Vasconcellos AS. Behavioural and hormonal effects of member replacement in captive groups of blue-fronted amazon parrots (Amazona aestiva). Behavioural Processes. 2017;138:160-9.https://doi.org/10.1016/j.beproc.2017.03.006
67. Lievin-Bazin A, Pineaux M, Le Covec M, Gahr M, Bovet D, von Bayern AMP. Food sharing and affiliation: An experimental and longitudinal study in cockatiels (Nymphicus hollandicus). Ethology. 2019;125(5):276-88.https://doi.org/10.1111/eth.12850
68. Aengus WL, Millam JR. Taming parent-reared orange-winged Amazon parrots by neonatal handling. Zoo biology. 1999;18(3):177-87.https://doi.org/10.1002/(SICI)1098-2361(1999)18:3<177::AID-ZOO2>3.0.CO;2-D
69. Collette JC, Millam JR, Klasing KC, Wakenell PS. Neonatal handling of Amazon parrots alters the stress response and immune function. Applied Animal Behaviour Science. 2000;66(4):335-49.https://doi.org/10.1016/S0168-1591(99)00098-2
70. Miller ML, Gallup AC, Vogel AR, Clark AB. Handling stress initially inhibits, but then potentiates yawning in budgerigars (Melopsittacus undulatus). Animal Behaviour. 2010;80(4):615-9.https://doi.org/10.1016/j.anbehav.2010.05.018
71. Bertin A, Beraud A, Lansade L, Blache MC, Diot A, Mulot B, et al. Facial display and blushing: Means of visual communication in blue-and-yellow macaws (Ara Ararauna)? PloS one. 2018;13(8):e0201762.https://doi.org/10.1371/journal.pone.0201762
72. Burmeister A-K, Drasch K, Rinder M, Prechsl S, Peschel A, Korbel R, et al. Development and Application of the Owner-Bird Relationship Scale (OBRS) to Assess the Relation of Humans to Their Pet Birds. Frontiers in Veterinary Science. 2020;7.https://doi.org/10.3389/fvets.2020.575221
73. Bertin A, Mulot B, Nowak R, Blache M-C, Love S, Arnold M, et al. Captive Blue-and-yellow macaws (Ara ararauna) show facial indicators of positive affect when reunited with their caregiver. Behavioural Processes. 2023;206:104833.https://doi.org/10.1016/j.beproc.2023.104833
74. Bertin A, Beraud A, Lansade L, Mulot B, Arnould C. Bill covering and nape feather ruffling as indicators of calm states in the Sulphur-crested cockatoo (Cacatua galerita). Behav Processes. 2020;178:104188.https://doi.org/10.1016/j.beproc.2020.104188
75. Bavelaar FJ, Beynen AC. Severity of atherosclerosis in parrots in relation to the intake of alpha-linolenic acid. Avian Dis. 2003;47(3):566-77.https://doi.org/10.1637/6052
76. Pereira LQ, Strefezzi Rde F, Catão-Dias JL, Trindade MM, Fighera RA, Kommers GD, et al. Hepatic hemosiderosis in red-spectacled Amazons (Amazona pretrei) and correlation with nutritional aspects. Avian Dis. 2010;54(4):1323-6.https://doi.org/10.1637/9418-060710-Case.1
77. Beaufrère H, Ammersbach M, Reavill DR, Garner MM, Heatley JJ, Wakamatsu N, et al. Prevalence of and risk factors associated with atherosclerosis in psittacine birds. Journal of the American Veterinary Medical Association. 2013;242(12):1696-704.https://doi.org/10.2460/javma.242.12.1696
78. Beaufrère H, Vet DM, Cray C, Ammersbach M, Tully TN. Association of Plasma Lipid Levels With Atherosclerosis Prevalence in Psittaciformes. Journal of Avian Medicine and Surgery. 2014;28(3):225-31, 7.https://doi.org/10.1647/2013-030
79. Rosenwax AC, Cowan ML. Fibrous ingluvial foreign bodies in 33 cockatiels (Nymphicus hollandicus). Australian veterinary journal. 2015;93(10):381-4.https://doi.org/10.1111/avj.12367
80. Beaufrère H, Reavill D, Heatley J, Susta L. Lipid-Related Lesions in Quaker Parrots (Myiopsitta monachus). Veterinary Pathology. 2019;56(2):282-8.https://doi.org/10.1177/0300985818800025

81. Gibson DJ, Nemeth NM, Beaufrère H, Varga C, Eagalle T, Susta L. Captive psittacine birds in Ontario, Canada: a 19-year retrospective study of the causes of morbidity and mortality. Journal of Comparative Pathology. 2019;171:38-52.https://doi.org/10.1016/j.jcpa.2019.07.002
82. Berg ML, Knott B, Ribot RFH, Buchanan KL, Bennett ATD. Do glucocorticoids or carotenoids mediate plumage coloration in parrots? An experiment in Platycercus elegans. Gen Comp Endocrinol. 2019;280:82-90.https://doi.org/10.1016/j.ygcen.2019.04.014
83. Vendramin-Gallo M, Pessutti C, Pezzato AC, Vicentini-Paulino ML. Effect of age on seed digestion in parrots (Amazona aestiva). Physiological and biochemical zoology : PBZ. 2001;74(3):398-403.https://doi.org/10.1086/320418
84. Larcombe SD, Tregaskes CA, Coffey J, Stevenson AE, Alexander LG, Arnold KE. Oxidative stress, activity behaviour and body mass in captive parrots. Conservation physiology. 2015;3(1):cov045.https://doi.org/10.1093/conphys/cov045
85. Owen DJ, Lane JM. High levels of corticosterone in feather-plucking parrots (Psittacus erithacus). Veterinary Record. 2006;158(23):804-5.https://doi.org/10.1136/vr.158.23.804
86. Cabezas S, Carrete M, Tella JL, Marchant TA, Bortolotti GR. Differences in acute stress responses between wild-caught and captive-bred birds: a physiological mechanism contributing to current avian invasions? Biological Invasions. 2013;15(3):521-7.https://doi.org/10.1007/s10530-012-0304-z
87. Costa P, Macchi E, Valle E, De Marco M, Nucera DM, Gasco L, et al. An association between feather damaging behavior and corticosterone metabolite excretion in captive African grey parrots (Psittacus erithacus). PeerJ. 2016;4.https://doi.org/10.7717/peerj.2462
88. Vidal AC, Roldan M, Christofoletti MD, Tanaka Y, Galindo DJ, Duarte JMB. Stress in captive Blue-fronted parrots (Amazona aestiva): the animalists' tale. Conservation physiology. 2019;7(1):coz097.https://doi.org/10.1093/conphys/coz097
89. West JA, Tully TN, Nevarez JG, Stout RW. Effects of fluorescent lighting versus sunlight exposure on calcium, magnesium, vitamin D, and feather destructive behavior in Hispaniolan Amazon parrots (Amazona ventralis). Journal of Avian Medicine and Surgery. 2019;33(3):235-44.https://doi.org/10.1647/2018-378
90. Nightengale M, Stout RW, Tully TN. Plasma Vitamin D (25-Hydroxyvitamin D) Levels in Hispaniolan Amazon Parrots (Amazona ventralis) Housed Indoors Over Time. Avian Dis. 2022.https://doi.org/10.1637/aviandiseases-D-21-00117
91. Aydinonat D, Penn DJ, Smith S, Moodley Y, Hoelzl F, Knauer F, et al. Social isolation shortens telomeres in African Grey parrots (Psittacus erithacus erithacus). PloS one. 2014;9(4):e93839.https://doi.org/10.1371/journal.pone.0093839
92. Dos Santos GJ, Aleixo ASC, Hippólito AG, Ferro BS, Okamoto P, Lourenço MLG, et al. Are echocardiographic parameters affected by body condition scores in blue-fronted Amazon parrots (Amazona aestiva, Linnaeus, 1758)? Veterinary research communications. 2022.https://doi.org/10.1007/s11259-022-09894-8
